# Identification and expression analysis of strigolactone biosynthetic and signaling genes reveal strigolactones are involved in fruit development of the woodland strawberry (*Fragaria vesca*)

**DOI:** 10.1186/s12870-019-1673-6

**Published:** 2019-02-14

**Authors:** Han Wu, Huihui Li, Hong Chen, Qi Qi, Qiangqiang Ding, Juan Xue, Jing Ding, Xiangning Jiang, Xilin Hou, Yi Li

**Affiliations:** 1 State Key Laboratory of Crop Genetics and Germplasm Enhancement, College of Horticulture, Nanjing Agricultural University, Nanjing, 210095 China; 2 Jiangsu Key Laboratory for the Research and Utilization of Plant Resources, Institute of Botany, Jiangsu Province and Chinese Academy of Sciences, Nanjing, 210014 China; 3 National Engineering Laboratory for Tree Breeding, College of Life Sciences and Biotechnology, Beijing Forestry University, Beijing, 100083 China; 4 Department of Plant Science and Landscape Architecture, University of Connecticut, Storrs, CT 06269 USA; 5 Present address: Fuyang Academy of Agricultural Sciences, Fuyang, 236065 China

**Keywords:** Woodland strawberry, Genome-wide identification, Expression analysis, Biosynthetic and signaling genes, Strigolactones, Fruit development

## Abstract

**Background:**

The development and ripening of fresh fruits is an important trait for agricultural production and fundamental research. Almost all plant hormones participate in this process. Strigolactones (SLs) are a new class of plant hormones that regulate plant organ development and stress tolerance, but little is known about their roles in fruit development.

**Results:**

In this study, we identified SL biosynthetic and signaling genes in woodland strawberry, a typical non-climacteric fruit, and analyzed the expression patterns of these genes in different plant tissues and developing fruits. One *D27*, two *MAX1*, and one *LBO* gene were identified as involved in SL biosynthesis, and one *D14*, one *D3*, and two *D53* genes as related to SL signaling. The proteins encoded by these genes had similar motifs as SL biosynthetic and signaling proteins in rice and *Arabidopsis*. The genes had different expression levels in the root, stem, leaf, and petiole of woodland strawberry. In addition, the expression of most SL biosynthetic genes was high in developing carpel, anther, and style, while that of SL signaling genes was high in carpel and style, but low in anther, suggesting active SL biosynthesis and signaling in the developing carpel and style. Notably, the expression of SL biosynthetic and signaling genes was significantly increased in the receptacle after pollination and decreased during receptacle development. Moreover, low or no expression of these genes was detected in ripening fruits.

**Conclusions:**

Our results suggest that SLs play a role in the early stages of woodland strawberry fruit development. Our findings provide insight into the function of SLs and will facilitate further study of the regulation by SLs of fresh fruit development.

**Electronic supplementary material:**

The online version of this article (10.1186/s12870-019-1673-6) contains supplementary material, which is available to authorized users.

## Background

The fresh fruit provides an ample and reliable source of food for people. The majority of plant hormones—including auxin, cytokinins (CKs), gibberellins (GAs), brassinolide (BR), jasmonic acid (JA), salicylic acid (SA), abscisic acid (ABA), and ethylene—participate in the development and ripening of fresh fruits [1–3]. However, the role of strigolactones (SLs), which were accepted as a new type of plant hormones in 2008 [4, 5], in the development of fresh fruits is unclear.

SLs are a small group of strigol-type compounds. The first natural SL, strigol, was isolated from cotton root exudates as a potent seed germination stimulant of the root-parasitized weed *Striga lutea* [6]. Other SLs have been identified in diverse plant species [7]. In 2008, SLs were found to have the ability of inhibiting plant branching, so they were accepted as a new type of plant hormones that modulate plant development [4, 5]. The physiological functions of SLs have been widely excavated in the past decade, and several genes involved in their biosynthetic and signaling pathways have been identified [8, 9].

SLs are carotenoid derivatives belonging to terpene lactones, and five major components have been identified in SL biosynthetic pathway: isomerase DWARF27 (D27), Carotenoid Cleavage Dioxygenase 7 (CCD7), Carotenoid Cleavage Dioxygenase 8 (CCD8), cytochrome P450 monooxygenase MORE AXILLARY GROWTH 1 (MAX1), and oxidoreductase-like enzyme LATERAL BRANCHING OXIDOREDUCTASE (LBO) [9, 10]. First, *trans*-β-carotene is catalyzed by D27 to produce 9-*cis*-β-carotene, which is cleaved by CCD7 into 9-cis-β-apo-10′-carotenal. Next, CCD8 converts this cleavage product into carlactone (CL), the biosynthetic precursor for all known type of SLs. The biosynthesis of CL from *trans*-β-carotene is conserved, while the pathway from CL to active SLs varies among plant species. In *Arabidopsis*, CL is converted by MAX1 to carlactonoic acid (CLA) via 19-hydroxy-carlactone. CLA is subsequently converted into methyl carlactonoate (MeCLA) by an unknown methyl transferase. MeCLA has some activity in shoot branching suppression, a classic SL role, but LBO can further convert MeCLA into an unidentified SL-like compound that may be the final product of SL biosynthesis and have more SL activity in *Arabidopsis*. In rice, CL is converted into CLA by the MAX1 homolog Os900 (CYP711A2), which also catalyzes the conversion of CLA to 4-deoxyorobanchol (4DO). Subsequently, a second rice MAX1 homolog, Os1400 (CYP711A3), converts 4DO into orobanchol, which has SL activity. In addition, CL is assumed to be the precursor of 5-deoxystrigol, which can be converted into sorgomol (a sorghum strigol-like SL), although evidence for this is limited [9]. The genes encoding D27, CCD7, CCD8, MAX1, and LBO have been identified in several plant species, such as *Arabidopsis D27*/rice *D27* [11, 12], *Arabidopsis MAX3*/rice *HTD1*/pea *RMS5*/petunia *DAD3/*tomato *CCD7* [13–17], *Arabidopsis MAX4*/rice *D10*/pea *RMS1*/petunia *DAD1/*tomato *CCD8* [18–22], *Arabidopsis MAX1*/rice *MAX1s*/petunia *MAX1* [23–25], and *Arabidopsis LBO* [10]. Mutants in these genes display an increased number of tillers or branches and a dwarf phenotype; both can be rescued by SL treatment. However, several components of the SL biosynthesis pathway remain to be identified [9].

One SL signaling pathway has been investigated extensively in *Arabidopsis* and rice. This pathway has five major components: D14, D3/MAX2, D53/SMXLs, TPR2, and IPA1 [9, 26]. The SL receptor D14 is an α/β hydrolase [27–29], and D3 is an F-box protein that forms a ubiquitination complex with an SCF-type ubiquitin ligase [30, 31]. D53 has been identified as a key transcriptional repressor in SL signaling [32–34]. In the absence of SLs, D14 cannot interact with D3 and D53, while D53 can interact with the transcriptional co-repressor TOPLESS (TPL)-related protein TPR2 and the transcription factor Ideal Plant Architecture1 (IPA1) to repress the transcriptional activity of IPA1. The end result is suppression of the expression of downstream IPA1-regulated genes and no SL response, such as increased tiller or branches. Inversely, In the presence of SLs, D14 binds and hydrolyzes SL molecules, triggering a conformational change in D14 to form a complex with D3 and D53, resulting in D53 degradation via the SCF^D3^ ubiquitination complex, then IPA1 cannot form the complex in the D53- and TPR2-dependent process, resulting in expression of IPA1-regulated genes and an SL response [26, 35].

Prior work on SLs has focused on their roles in *Arabidopsis* and rice in morphogenesis, root structure, shoot branching, stem elongation, leaf morphology, and stress resistance [8, 9, 36]. However, little is known about the role of SLs in fresh fruit development. Cultivated strawberry is an octaploid non-climacteric fresh fruit, and the complexity of its genome hampers molecular, genetic, and functional studies. The ease of genetic transformation of diploid strawberries, particularly woodland strawberry (*Fragaria vesca*), has led to their use in the study of rosaceae plants and non-climacteric fresh fruits [37]. In this study, we identified genes involved in SL biosynthesis and signaling, and analyzed their expression patterns in woodland strawberry vegetative organs and fruits at different developmental stages. The results suggested that SLs play a role in the development of woodland strawberry flower and immature fruit. Our findings will facilitate further studies of the relationship between SLs and the development of strawberry fruits.

## Results

### Identification of SL biosynthetic genes *D27*, *MAX1*, and *LBO* in woodland strawberry

**Fig. 1:**
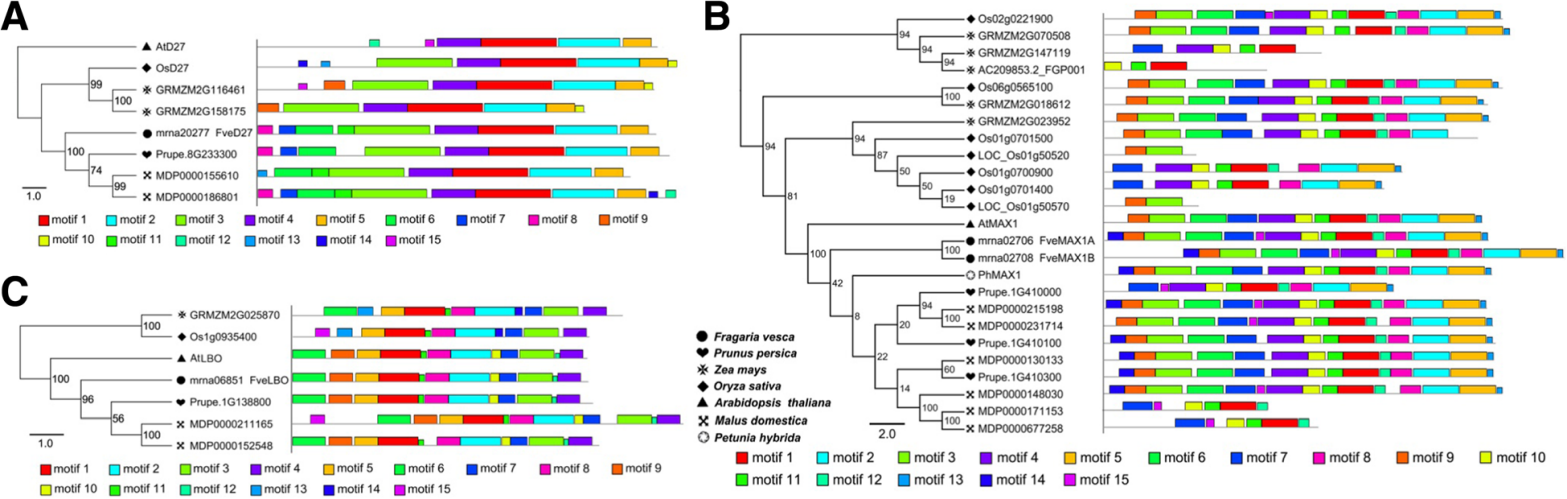
Conserved motif analysis of D27, MAX1 and LBO protein families from rice, *Arabidopsis*, apple, peach, maize, petunia, and woodland strawberry. **a** The motif composition of D27 family. **b** The motif composition of MAX1 family. **c** The motif composition of LBO family. Motifs were identified using MEME software, up to 15 motifs were permitted and other parameters were at the default settings. The 15 motifs were indicated by boxed of different color. Gray lines represent non-conserved sequences. Distribution and protein sequences of conserved motifs were presented in Additional files 2 and 3. rice (*Oryza sativa*), *Arabidopsis* (*Arabidopsis thaliana*)*,* apple (*Malus domestica*), peach (*Prunus persica*), maize (*Zea mays*), woodland strawberry (*Fragaria vesca*), and petunia (*Petunia hybrida*)

D27 is a 9-cis/all-trans-β-carotene isomerase and has been functionally analyzed in rice and *Arabidopsis* [11, 12]. We used the amino acid sequences of the rice and *Arabidopsis* D27 proteins as queries to identify similar proteins in rice, *Arabidopsis*, apple, peach, maize, and woodland strawberry protein databases, and constructed a phylogenetic tree. The identified proteins clustered into clades 1, 2, and 3. Clades 1 and 2 together formed a larger group with a probability of 99% (Additional file 1). Rice OsD27 and *Arabidopsis* AtD27 belonged to clade 1, which we named the D27 family. Interestingly, the clade 1/D27 family included one rice protein (OsD27), one *Arabidopsis* protein (AtD27), and one peach protein (Prupe.8G233300), but two apple proteins (MDP0000186801 and MDP0000155610) and two maize proteins (GRMZM2G116461 and GRMZM2G158175). Apple MDP0000186801 and MDP0000155610 clustered together with a bootstrap value of 99, as did maize GRMZM2G116461 and GRMZM2G158175 with a bootstrap value of 100 (Additional file 1; Fig. 1a), suggesting that *D27* underwent duplication in apple and maize during evolution. In woodland strawberry, only mrna20277 clustered in clade 1/D27, with a bootstrap value of 99 (Fig. 1a), suggesting that it participates in SL biosynthesis. In addition, a motif analysis using MEME showed that the N-terminal motifs of the proteins differed among plant species, including between rice OsD27 and *Arabidopsis* AtD27. However, the C-terminus of both rice OsD27 and *Arabidopsis* AtD27 harbors motifs 1, 2, 4, and 9, which may be important for D27 activity. The D27 proteins in apple, peach, maize, and woodland strawberry also contain these four motifs (Fig. 1a; Additional file 2; Additional file 3). Therefore, we named mrna20277 of woodland strawberry as FveD27.

SL biosynthetic genes *FveCCD7* and *FveCCD8* of woodland strawberry have been identified in a previous study [38], as the downstream component of *CCD7* and *CCD8*, the *MAX1* gene family was identified in *Arabidopsis*, rice, woodland strawberry, maize, apple, and peach in our study. MAX1, a cytochrome P450 monooxygenase, participates in the biosynthesis of SLs. Cytochrome P450 proteins can be divided into 10 clans: CYP71, CYP72, CYP85, CYP86, CYP51, CYP74, CYP97, CYP710, CYP711, and CYP727. SL biosynthetic gene *MAX1* belongs to the CYP711 clan [39] (Additional file 4). In this study, two woodland strawberry proteins (mrna02706 and mrna02708), three peach proteins (1G410000, 1G410100, and 1G410300), five maize proteins (GRMZM2G070508, GRMZM2G147119, AC209853.2_FGP001, GRMZM2G018612, and GRMZM2G023952), and six apple proteins (MDP0000215198, MDP0000231714, MDP0000130133, MDP0000148030, MDP0000171153, and MDP0000677258) clustered in CYP711 together with *Arabidopsis* AtMAX1 and petunia PhMAX1, which are related to SL biosynthesis (Fig. 1b). In addition, seven rice proteins (Os02g0221900, Os06g0565100, Os01g0701500, LOC_Os01g50520, Os01g0700900, Os01g0701400, and LOC_Os01g50570) clustered in CYP711 (Fig. 1b), but only Os01g0700900 (Os900) and Os01g0701400 (Os1400) are involved in SL biosynthesis [24]. A motif composition analysis showed that most of the proteins (including all of the functional analyzed MAX1 proteins in *Arabidopsis*, petunia, and rice) in CYP711 contain 9–15 motifs. However, GRMZM2G147119, AC209853.2_FGP001, LOC_Os01g50520, LOC_Os01g50570, MDP0000171153, and MDP0000677258 contain 2–6 motifs, suggesting that these six proteins do not have MAX1 activity. In woodland strawberry, mrna02706 and mrna02708 have the same motif composition composed by 15 motifs, similar to *Arabidopsis AtMAX1* and petunia *PhMAX1* (Fig. 1b; Additional file 2; Additional file 3). Therefore, mrna02706 and mrna02708 in woodland strawberry are likely orthologs of MAX1 and participate in SL biosynthesis; we named these proteins FveMAX1A and FveMAX1B, respectively.

*LBO*, which encodes an oxidoreductase-like enzyme, acts in the final stages of SL biosynthesis in *Arabidopsis*. LBO belongs to the DOXC54 clade of the 2-oxoglutarate and Fe(II)-dependent dioxygenase superfamily [10] (Additional file 5). We used the amino acid sequence of *Arabidopsis* LBO as a query to identify similar proteins in *Arabidopsis*, rice, wood strawberry, maize, apple, and peach, and constructed a phylogenetic tree using PhyML. The tree showed that LBO is highly conserved, one protein of *Arabidopsis* (LBO), rice (Os01g0935400), woodland strawberry (mrna06851), maize (GRMZM2G025870), and peach (1G138800), and two proteins (MDP0000211165 and MDP0000152548) of apple were included in the DOXC54 clade. MDP0000211165 and MDP0000152548 clustered together with a bootstrap value of 100 (Additional file 5), suggesting gene duplication. Further, woodland strawberry mrna06851, peach 1G138800, and apple MDP0000152548 have identical motif composition composed by 12 motifs as *Arabidopsis* LBO, while maize GRMZM2G025870, rice Os01g0935400, and apple MDP0000211165 have the following differences at the N-terminal: GRMZM2G025870 has motifs 13 and 14 instead of motifs 9, 10, and 12; Os01g0935400 has motifs 13–15 instead of motifs 6, 9, 10, and 12; and MDP0000211165 has one additional motif (motif 15) (Fig. 1c; Additional file 2; Additional file 3). The homologous genes in rice, woodland strawberry, maize, apple, and peach have not been studied, and so we named mrna06851 of woodland strawberry FveLBO.

### Identification of the SL receptor D14 in woodland strawberry

**Fig. 2:**
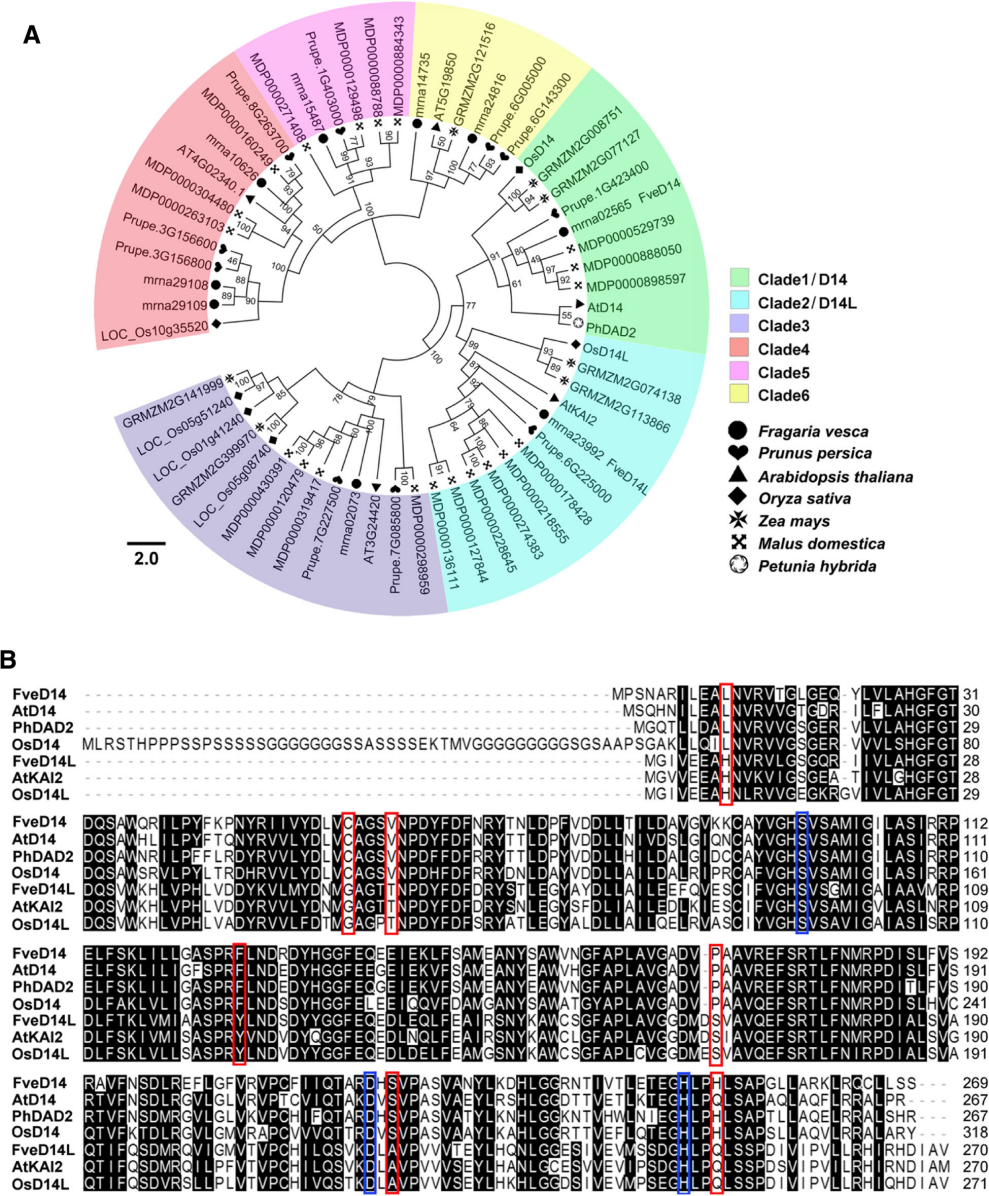
Maximum likelihood phylogeny and sequence alignment of D14 family. **a** Phylogenetic tree of D14 family identified from rice (*Oryza sativa*), *Arabidopsis* (*Arabidopsis thaliana*), apple (*Malus domestica*), peach (*Prunus persica*), maize (*Zea mays*), petunia (*Petunia hybrida*), and woodland strawberry (*Fragaria vesca*). **b** Sequence alignment of predicted D14 and D14L proteins of woodland strawberry (Fve) with the functionally analyzed D14 and D14L proteins of rice (Os), *Arabidopsis* (At), and petunia (Ph). The putative hydrolase catalytic triad residues of D14 and D14L were highlighted in blue boxes, and the seven specificity-determining positions (SDPs) which determine the functions of D14 and D14L proteins were highlighted in red boxes

*D14/DAD2*, which encodes an α/β hydrolyzyme, is the SL receptor gene in the plant SL signal transduction pathway [27–29]. We used the sequences of the D14 proteins from rice (OsD14), *Arabidopsis* (AtD14), and petunia (PhDAD2) with known function as queries to identify homologs in rice, *Arabidopsis*, maize, apple, peach, and woodland strawberry, and constructed a phylogenetic tree (Fig. 2a). The phylogenetic tree was divided into six clades: Clade1-Clade6. Clade1 and 2 clustered in a large group, with a bootstrap value of 77. The SL receptors OsD14, AtD14, and PhDAD2 were clustered into clade 1, and the KAR receptors OsD14L and AtKAI2 into clade 2, so we named clades 1 and 2 the D14 and D14L families, respectively. D14 family comprised one protein from rice (OsD14), *Arabidopsis* (AtD14), petunia (PhDAD2), woodland strawberry (mrna02565), and peach (Prupe.1G423400), two proteins from maize (GRMZM2G008751 and GRMZM2G077127), and three from apple (MDP0000529739, MDP0000888050, and MDP0000898597) (Fig. 2a). Similarly, D14L family comprised one rice protein (OsD14L), one *Arabidopsis* protein (AtKAI2), one woodland strawberry protein (mrna02565), one peach protein (Prupe.6G225000), two maize proteins (GRMZM2G074138 and GRMZM2G113866), and six apple proteins (MDP0000178428, MDP0000218555, MDP0000274383, MDP0000228645, MDP0000127844, and MDP0000136111) (Fig. 2a). Therefore, we named mrna02565 and mrna23992 as FveD14 and FveD14L of woodland strawberry, respectively. Interestingly, D14 and D14L proteins of maize and apple clustered together with a high bootstrap value, indicating the occurrence of gene duplications.

To investigate functional conservation during evolution, we analyzed motif composition using MEME. The motif composition was conserved within each clade, but divergent among different clades (Additional file 2; Additional file 3; Additional file 6). D14, D14L, and clade 3 have relatively similar motif composition compared to Clade4, Clade5 and Clade6. The majority of proteins in D14 and D14L have identical motif compositions, while most clade 3 proteins lack motif 3 compared to D14 and D14L families, suggesting involvement of motif 3 in binding SLs and KAR. In addition, apple MDP0000218555, MDP0000274383, and MDP0000228645 in D14L, and MDP0000120479 and MDP0000430391 in clade 3, are truncated proteins with four or five motifs, and so may have functions different from the other D14L and clade 3 proteins (Additional file 2; Additional file 3; Additional file 6). From the result, the identical motif composition of D14 and D14L proteins hampered determination of their functions.

In order to further study the difference of conserved amino acid sequences between D14 and D14L, We next performed a ClustalW multi-sequence alignment of D14 proteins (OsD14, AtD14, PhDAD2, and FveD14) and D14L proteins (AtKAI2, OsD14L, and FveD14L) (Fig. 2b). At the amino acid level, the putative hydrolase catalytic triad residues of FveD14 (Ser98-Asp219-His248) and FveD14L (Ser95-Asp217-His246) were identical to those of AtD14 (Ser97-Asp218-His247), OsD14 (Ser147-Asp268-His297), PhDAD2 (Ser96-Asp217-His246), AtKAI2 (Ser95-Asp217-His246), and OsD14L (Ser96-Asp218-His247). This suggests that the putative SL receptor FveD14 and the KAR receptor FveD14L from woodland strawberry have hydrolase activity. Seven specificity-determining positions (SDPs) determine the functions of D14- and D14L-related proteins. In our results, the seven SDPs of FveD14 (L11-C56-V60-F127-P170-S221-H251) were identical to those of other known functional D14 proteins (rice OsD14, *Arabidopsis* AtD14, and petunia PhDAD2), and SDPs of FvD14L (H8-G53-T57-Y124-S168-A219-Q249) were identical to those of other known D14L proteins (rice OsD14L and *Arabidopsis* AtKAI2) (Fig. 2b). These findings confirm that FveD14 and FveD14L are putative SL and KAR receptors, respectively, in woodland strawberry.

### Identification of *D3* and *D53* SL signaling genes in woodland strawberry

**Fig. 3:**
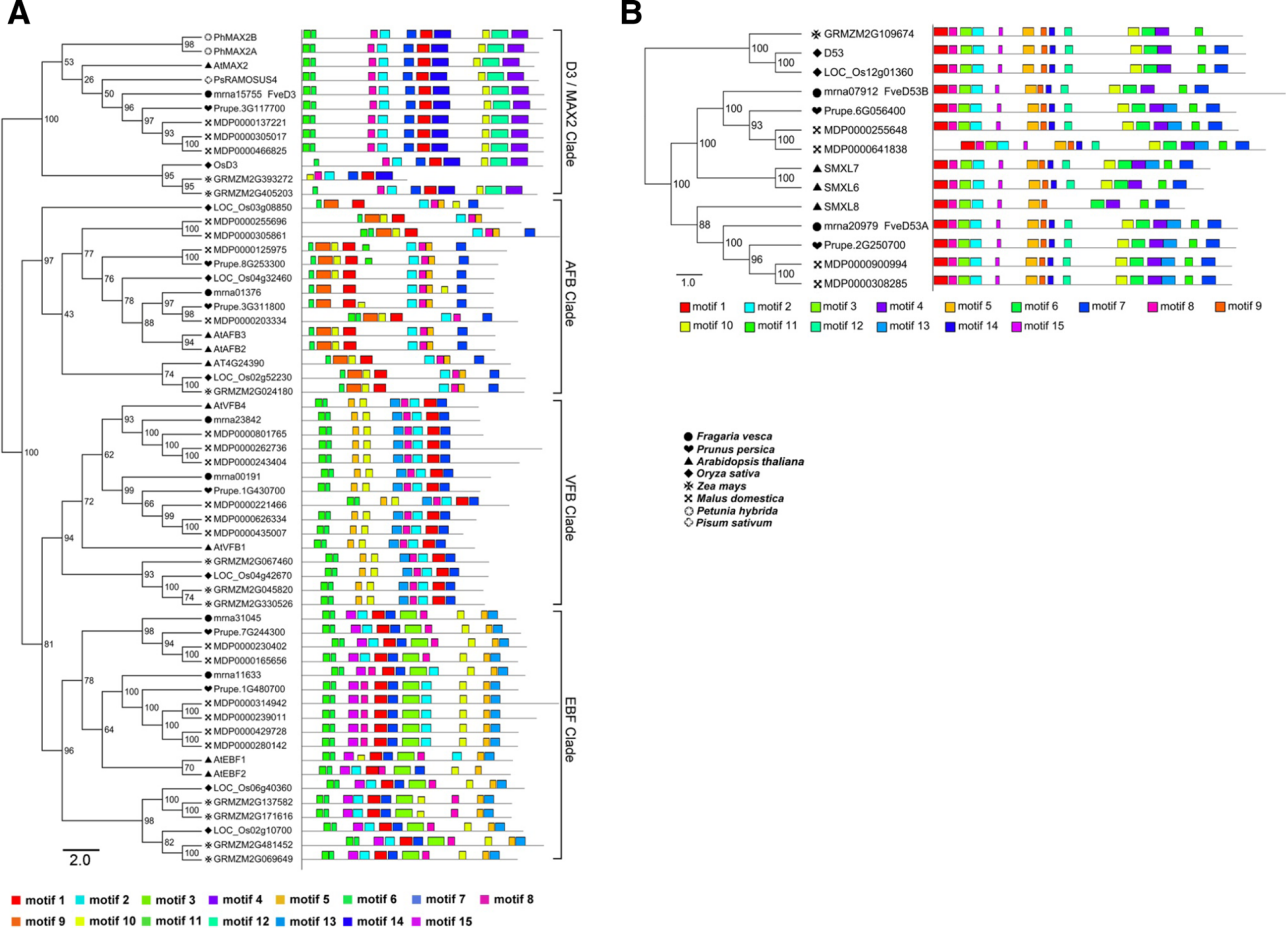
Conserved motif analysis of D3 and D53 protein families from rice, *Arabidopsis*, apple, peach, maize, petunia, pea, and woodland strawberry. **a** The motif composition of D3/MAX2, AFB, VFB, and EBF families. **b** The motif composition of D53 family. Motifs were identified using MEME software, up to 15 motifs were permitted and other parameters were at the default settings. The 15 motifs were indicated by boxed of different color. Gray lines represent non-conserved sequences. Distribution and protein sequences of conserved motifs were presented in Additional files 2 and 3. rice (*Oryza sativa*), *Arabidopsis* (*Arabidopsis thaliana*)*,* apple (*Malus domestica*), peach (*Prunus persica*), maize (*Zea mays*), woodland strawberry (*Fragaria vesca*), petunia (*Petunia hybrida*), pea (*Pisum sativum*)

*D3/MAX2* gene encodes an F-box leucine-rich repeat (LRR) protein that participates in SL signaling [30, 31]. The F-box protein family is a superfamily, and D3 proteins belong to the F-box LRR_7 family [40]. *D3* genes have been identified in rice (*OsD3*), *Arabidopsis* (*AtMAX2*), pea (*PsRMS4*), and petunia (*PhMAX2A*, *PhMAX2B*) [25, 30, 41, 42]. According to the phylogenetic tree, one woodland strawberry protein (mrna15755), one peach protein (3G117700), three apple proteins (MDP0000137221, MDP0000305017, and MDP0000466825), and two maize proteins (GRMZM2G393272 and GRMZM2G405203) clustered with OsD3, AtMAX2, PsRMS4, PhMAX2A, and PhMAX2B (Additional file 7). We next analyzed the motif composition of four F-box LRR_7 protein families according to *Arabidopsis* (bootstrap value > 90) (Fig. 3a; Additional file 2; Additional file 3). These four clusters we named the MAX2/D3, AFB, VFB, and EBF clades. The motif composition was conserved within each clade, but widely divergent among the clades. In the MAX2/D3 clade, the D3 proteins of dicotyledons (*Arabidopsis*, pea, petunia, woodland strawberry, peach, and apple) were clustered together, and these proteins contain 10 motifs, while the D3 proteins of monocotyledons (rice and maize) which clustered together only contain nine motifs, with the exception of GRMZM2G393272. GRMZM2G393272 is a truncated protein that lacked three C-terminal motifs, and thus may have a different function. The motif structure of woodland strawberry mrna15755 is consistent with that of MAX2/D3 clade proteins (Fig. 3a; Additional file 2; Additional file 3), suggesting a role in SL signaling; we thus named mrna15755 as FveD3.

D53/SMXL6/SMXL7/SMXL8 acts as a repressor of SL signalling and its degradation is induced by SLs [32–34]. Using rice D53 as a query, we identified 84 proteins in rice, *Arabidopsis*, apple, peach, maize, and woodland strawberry after removing redundant proteins. The resulting phylogenetic tree showed that the 84 proteins clustered into the D53-like, SMAX1-like, and ClassIClp ATPase clades (Additional file 8). D53-like and SMAX1-like clustered together to form a larger group, with a bootstrap value of 97. The SL signaling proteins D53/SMXL6/SMXL7/SMXL8 belong to the D53-like family, which also contains two rice proteins (D53 and Os12g01360), three *Arabidopsis* proteins (SMXL6, SMXL7, and SMXL8), two woodland strawberry proteins (mrna20979 and mrna07912), one maize protein (GRMZM2G109674), two peach proteins (Prupe.6G056400 and Prupe.2G250700), and four apple proteins (MDP0000255648, MDP0000641838, MDP0000900994, and MDP0000308285) (Additional file 8). *Arabidopsis* SMXL7 contained 15 motifs, while SMXL6, SMXL8, and rice D53 lacked one, four, and one motif, respectively, but these four proteins all have been demonstrated as SL signaling proteins. Maize GRMZM2G109674 lacked motif 7 compared to rice D53; but all D53 homologs of peach and apple were consistent with *Arabidopsis* SMXL7. Interestingly, woodland strawberry mrna20979 has 15 motifs, consistent with *Arabidopsis* SMXL7, while mrna07912 has 14 motifs, consistent with *Arabidopsis* SMXL6 (Fig. 3b; Additional file 2; Additional file 3). Thus, the function of mrna20979 and mrna07912 is likely to be identical to that of D53, and so we named mrna20979 as FveD53A and mrna07912 as FveD53B.

### Expression of SL biosynthetic and signaling genes in woodland strawberry vegetative tissues

**Fig. 4:**
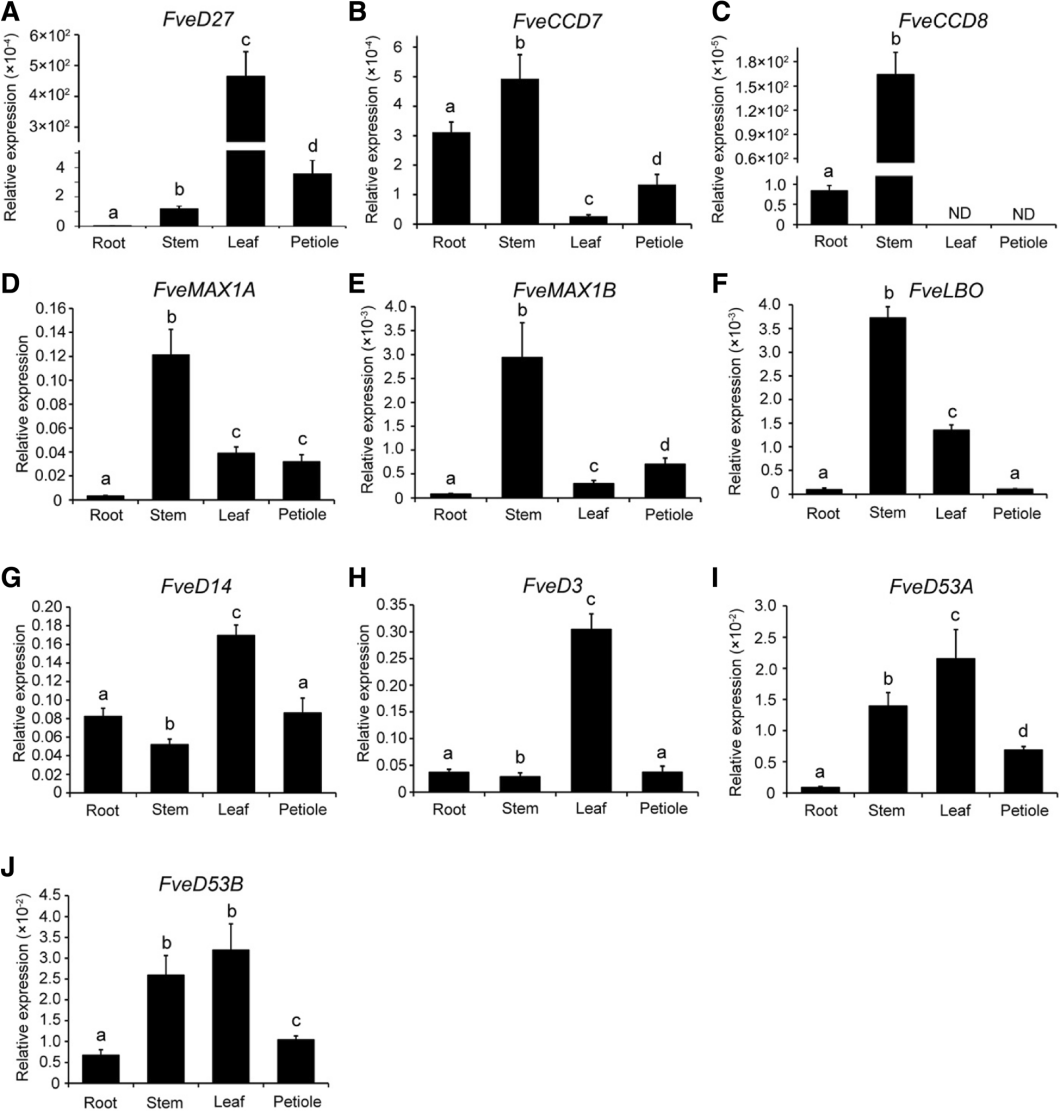
Expression analysis of SL biosynthetic and signaling genes in the root, stem, leaf and petiole of woodland strawberry. **a**-**j** Expression levels of SL biosynthetic genes *FveD27* (**a**), *FveCCD7* (**b**), *FveCCD8* (**c**), *FveMAX1A* (**d**), *FveMAX1B* (**e**), *FveLBO* (**f**), SL receptor gene *FveD14* (**g**) and signaling genes *FveD3* (**h**), *FveD53A* (**i**) and *FveD53B* (**j**). The transcript values were measured using qPCR with *GAPDH* as reference gene. Results were analyzed using the 2^-ΔCT^method.. Different letters at the top of each column indicate a significant difference at *P* < 0.05 (*n* = 3), by Student’s t-test

To assess the role of SLs in vegetative tissue growth and development of woodland strawberry, the expression levels of SL biosynthetic and signaling genes in the root, stem, leaf, and petiole of woodland strawberry were analyzed by qPCR. The result showed that, in strawberry seedlings, the expression of *FveD27* was low in root and slightly higher in stem and petiole, but *FveD27* expression in leaf was several hundred-fold higher than that in stem and petiole (Fig. 4a). *FveCCD7* was expressed mainly in root and stem, and at a low level in leaf; indeed, *FveCCD7* expression in stem was approximately nine-fold that in leaf, and four-fold higher in petiole than in leaf (Fig. 4b). The expression of *FveCCD8* was highest in stem, at approximately 150-fold that in root, but was not expressed in leaf or petiole (Fig. 4c). In addition, the expression of *FvMAX1A* and *FvMAX1B* was highest in stem, lowest in root, and moderate in leaf and petiole (Fig. 4d, e). *FveLBO* was highly expressed in stem, at approximately 2.7-fold that in leaf, and low expressed in root and petiole (Fig. 4f). For SL signaling genes, the transcript level of the SL receptor-encoding gene *FveD14* was highest in leaf, being threefold that in root, stem, and petiole (Fig. 4g), and the expression of *FveD3* in leaf was six-fold that in root, stem, and petiole (Fig. 4h). In addition, *FveD53A* and *FveD53B* had the highest expression in leaf, moderate in stem, and the lowest expression in root and petiole (Fig. 4i, j). To summarize, the putative SL biosynthetic genes in woodland strawberry showed the highest expression in stem, except for *FvD27*, while the putative SL signaling genes showed the highest expression levels in leaf. Therefore, SL biosynthesis and signal transduction activity may differ among the tissues of woodland strawberry plants.

### Expression of SL biosynthetic and signaling genes in woodland strawberry flower and early fruits

**Fig. 5:**
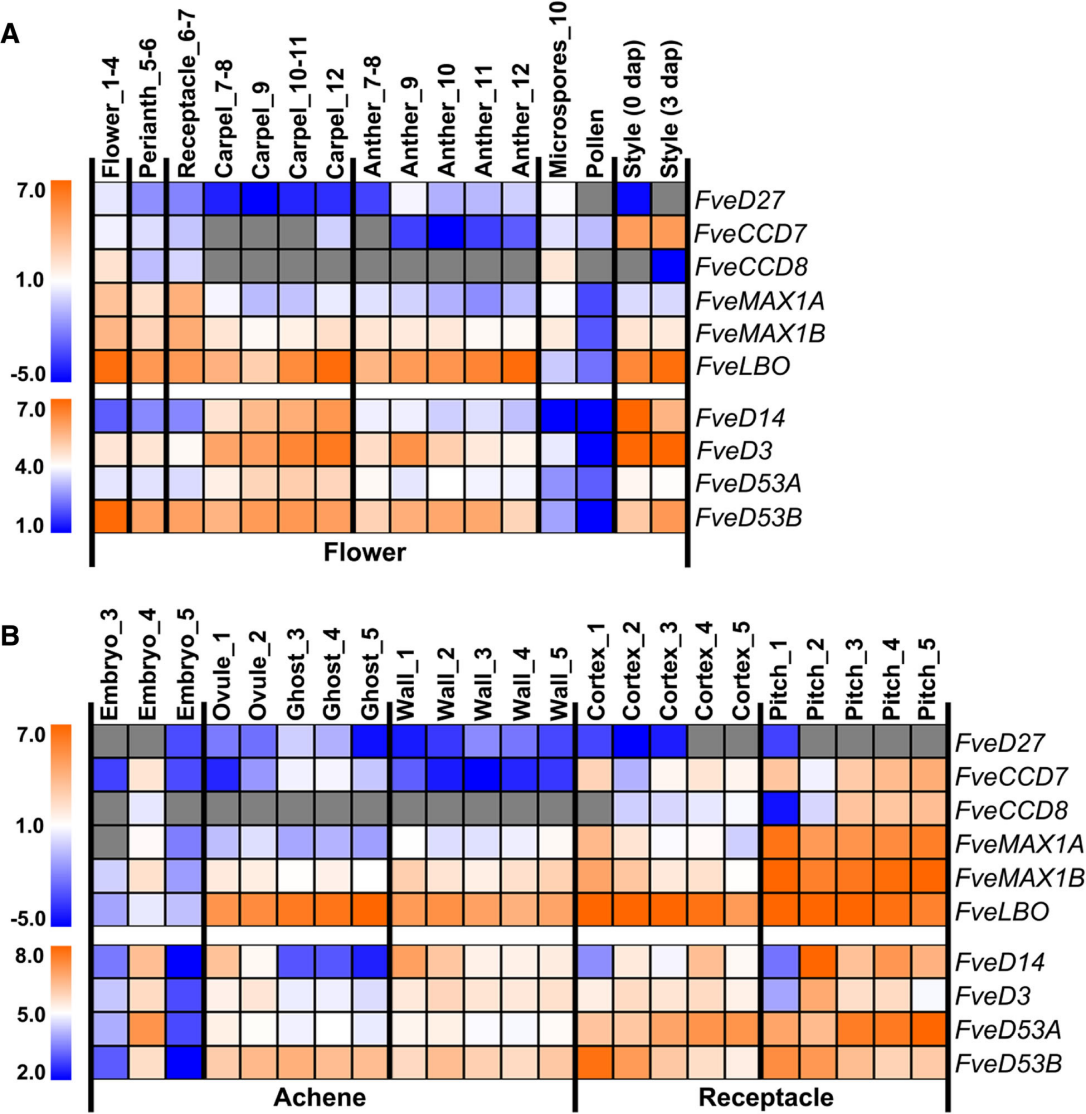
Expression pattern of SL biosynthetic and signaling genes in the flower and early-stage fruit of woodland strawberry. **a** Expression pattern of SL biosynthetic and signaling genes in the flower tissues (including perianth, carpel, anther, microspore, pollen, and style). **b** Expression pattern of SL biosynthetic and signaling genes in the early-stage of developing fruit tissues (including embryo, ovule, ghost, wall, cortex, and pith) of woodland strawberry. The transcriptome data was downloaded from (http://bioinformatics.towson.edu/strawberry/) [43]. RPKM values of related genes were in Additional file 9, and transformed in log2 level, and a heatmap was shown using MeV4.8 software. Gray boxes indicate the expression of genes was undetectable. Numbers after flower and fruit tissues represent the developmental stages of flowers and fruits, respectively. The detailed description of the stages and tissues was on the website (http://bioinformatics.towson.edu/strawberry/newpage/Tissue_Description.aspx)

To investigate the role of SL in woodland strawberry reproductive development, expression data of SL biosynthetic and signaling genes were obtained from Strawberry Genomic Resources (http://bioinformatics.towson.edu/strawberry/) [43] (Additional file 9), and are presented as heat maps. Strawberry flower undergoes 13 developmental stages, which are divided into three parts: stages 1–4, early flower development; stages 5–7, initiation and development of reproductive organs; and stages 8–13, differentiation of floral organs [44]. The expression levels of the putative SL biosynthetic and signaling genes differed among both tissue types and developmental stages (Fig. 5). The transcript levels of the SL biosynthetic genes *FveD27*, *FveCCD7*, *FveCCD8*, *FveMAX1A*, *FveMAX1B*, and *FveLBO* were generally higher in young complete flowers (1–4 flowered_B_C, 5–6 perianth, and 6–7 receptacle) compared to other mature flower tissues (7–12 carpels and anthers), but the transcript levels of the SL signaling genes *FveD14*, *FveD3*, and *FveD53A* were low (Fig. 5a). During carpel development, the transcript levels of *FveMAX1A*, *FveMAX1B*, and *FveLBO* initially decreased and subsequently increased. The transcript levels of *FvD27*, *FveCCD7*, and *FveCCD8* were very low or undetectable. By contrast, the transcript levels of the putative SL signaling genes *FveD14*, *FveD3*, *FveD53A*, and *FveD53B* were high in the developing carpel (Fig. 5a). During anther development, the transcript levels of SL biosynthetic genes were similar to those in the developing carpel*,* while those of the SL signaling genes were lower than in the developing carpel. In addition, the transcript levels of SL biosynthetic and signaling genes in anther decreased from stages 9–12, suggesting reduced SL activity (Fig. 5a). Interestingly, stage 10 microspores exhibited high and low transcript levels of SL biosynthetic and signaling genes, respectively, whereas the transcript levels of the biosynthetic and signaling genes were low in mature pollen. In addition, the transcript levels of *FveCCD7*, *FveAMX1A*, *FveMAX1B*, and *FveLBO* were high in style before and after pollination, but those of *FveD27* and *FveCCD8* were negligible. Notably, the transcript levels of *FveD14* and *FveD3* were high in style (Fig. 5a), suggesting active SL signaling before and after pollination.

Early fruit development of woodland strawberry from anthesis to green fruit is divided into five stages: stage 1 (pre-fertilization), stage 2 (2–4 days post-anthesis [DPA]), stage 3 (6–9 DPA), stage 4 (8–10 DPA), and stage 5 (10–13 DPA) [43]. The ovule and ovary wall at stages 1–2 showed high transcript levels of *FveMAX1A*, *FveMAX1B*, *FveLBO*, *FveD14*, *FveD3*, and *FveD53s*, while those of the SL biosynthetic genes *FveD27*, *FveCCD7*, and *FveCCD8* were low or undetectable. Achene at stages 3–5 was divided into embryo, ghost (seeds without embryos), and wall. The transcript levels of *FvMAX1A* and *FvMAX1B* in wall increased slightly from stages 3–5, while those of *FveLBO*, *FveD14*, *FveD3*, and *FveD53s* showed little or no change, and the transcript levels of *FveD27*, *FveCCD7*, and *FveCCD8* were low or negligible. In developing ghosts, the transcript levels of most biosynthetic and signaling genes were low, while those of *FveD27*, *FveCCD7*, and *FveLBO* were high in stage 3–4 ghosts (Fig. 5b). During embryonic development of early achene, the expression patterns of SL biosynthetic and signaling genes show distinct characteristics. Expression of all of the SL biosynthetic and signaling genes was low at embryo stage 3, but increased at embryo stage 4 (with the exception of *FvD27*) and decreased at embryo stage 5 (Fig. 5b), suggesting that SLs are important for embryonic development at stage 4. The receptacle was divided into the cortex and pith. The *FveD27* transcript level was low or undetectable in developing cortex and pith, but those of other SL biosynthetic genes were higher. In addition, the transcript levels of SL signaling genes were high in cortex and pith at stages 2–5. Notably, the transcript levels of *FveD14* and *FveD3* increased significantly after pollination in cortex and pitch (stages 1–2) (Fig. 5b), suggesting activation of SL signaling in receptacle due to pollination.

### Expression of SL biosynthetic and signaling genes during woodland strawberry fresh fruit development

**Fig. 6:**
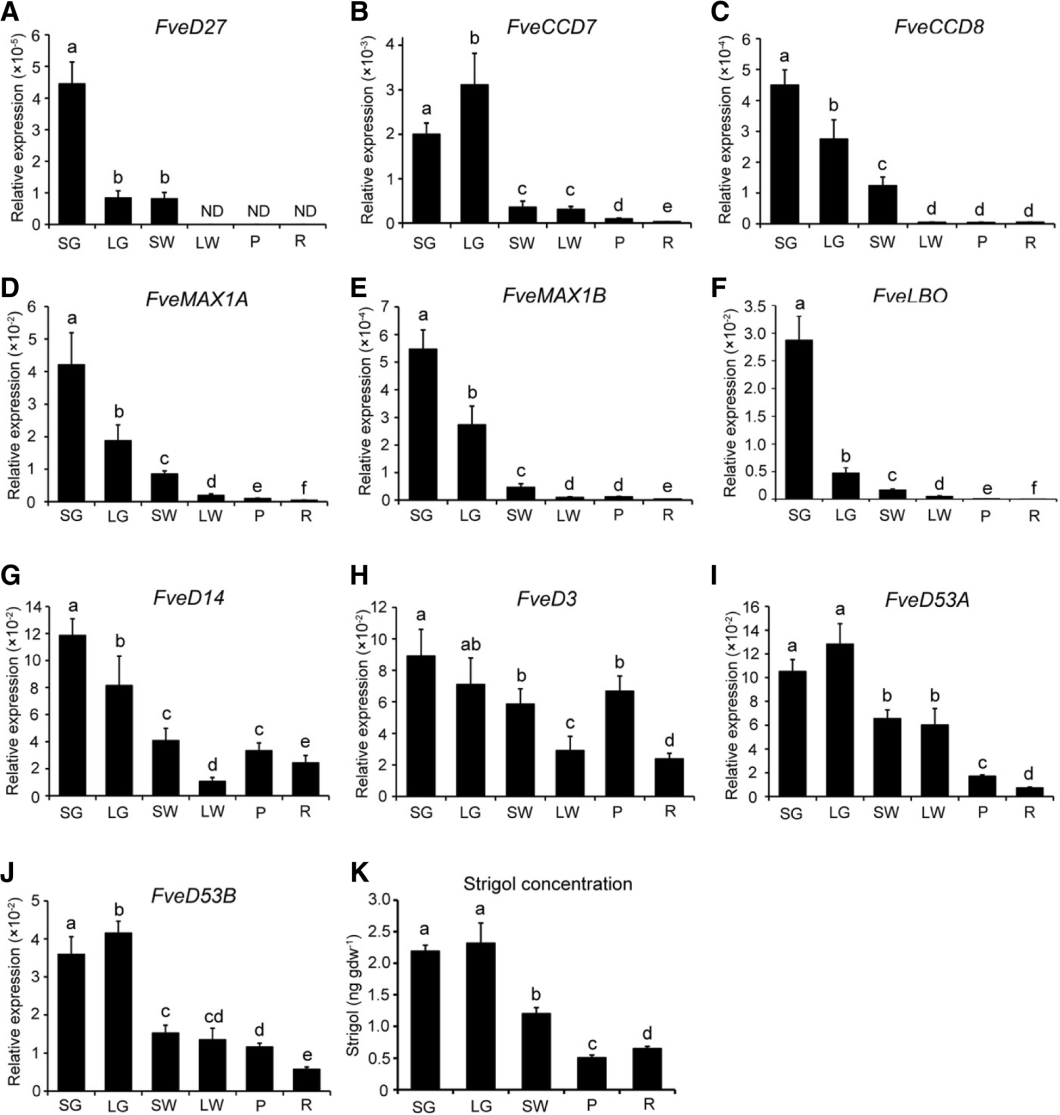
Expression analysis of SL biosynthetic and signaling genes, and measurement of strigol levels in the receptacle of woodland strawberry during fruit ripening. **a**-**j** Expression levels of SL biosynthetic genes *FveD27* (**a**), *FveCCD7* (**b**), *FveCCD8* (**c**), *FveMAX1A* (**d**), *FveMAX1B* (**e**), *FveLBO* (**f**), SL receptor gene *FveD14* (**g**) and signaling genes *FveD3* (**h**), *FveD53A* (**i**) and *FveD53B* (**j**), (**k**) High performance liquid chromatography coupled with electrospray ionization multi-tandem mass spectrometry (HPLC-ESI-MSn) measurement of strigol levels in the receptacle of woodland strawberry. Fruit samples were collected at the following six developmental stages: small green (SG, about 8 days post anthesis [DPA]), large green (LG, about 15 DPA), small white (SW, white pulp with green seeds, about 20 DPA), large white (LW, white pulp with red seeds, about 24 DPA), pink (P, slightly pink pulp with red seeds, about 26 DPA), and red (R, pulp all red, about 28 DPA) [38, 45, 46]. The transcript values were measured using qPCR with *GAPDH* as reference gene. Results were analyzed using the 2^-ΔCT^ method.. Different letters at the top of each column indicate a significant difference at *P* < 0.05 (*n* = 3), by Student’s t-test

To investigate the role of SLs in the development and ripening of woodland strawberry fruits, the expression of SL biosynthetic and signaling genes was analyzed by qPCR in receptacles lacking seeds at the small green (SG), large green (LG), small white (SW), large white (LW), pink (P), and red (R) stages [45, 46]. The result showed that the overall expression of *FveD27* in the receptacle was low, consistent with the transcriptome data. The expression of *FveD27* was highest in SG receptacle, approximately four-fold that in LG and SW receptacles. At the LW, P, and R stages, *FveD27* transcript was barely detected (Fig. 6a). The expression of *FveCCD7* increased from the SG stage, peaked at the LG stage, decreased significantly at the SW stage, and was very low at the LW, P, and R stages (Fig. 6b). *FveCCD8*, *FvMAX1A*, *FvMAX1B*, and *FveLBO* had similar expression profiles; their expression was highest at the SG stage, and gradually decreased to a low level at the LW, P, and R stages (Fig. 6c-f). Expression of the SL signaling genes *FvD14*, *FvD3*, *FveD53A*, and *FveD53B* was highest at the SG and LG stages, and decreased thereafter. Interestingly, unlike the SL biosynthetic genes, expression of the SL signaling genes was high at the ripening stages (LW, P, and R) (Fig. 6g-j). Indeed, the expression of *FveD14* and *FveD3* increased at the P stage, and decreased at the R stage (Fig. 6g, h). Further, we measured the concentration of strigol (one native SL) in woodland strawberry receptacle at five developmental stages (SG, LG, SW, P and R stages) by HPLC-ESI-MSn. The result showed that the concentration of strigol was also high at early stages (SG and LG), and then decreased from SW stage to ripening stages (P and R) (Fig. 6k). To summarize, the expression of SL biosynthetic and signaling genes is high in early developing fruits but decreases as fruit development and ripening proceed, consistent with SL concentrations, suggesting that SLs play a role in the early development of strawberry fruits.

## Discussion

### Identification of SL biosynthestic and signaling genes in woodland strawberry

SLs play important roles in plant growth and development, and their biosynthetic and signaling genes in *Arabidopsis* and rice have been investigated extensively*. Arabidopsis* and rice have one each of the *D27*, *CCD7*, *CCD8*, *D14*, and *D3* genes, which participate in SL biosynthesis and signaling [8, 9]. In woodland strawberry, there is also only one protein clustered together with *Arabidopsis* and rice D27, CCD7, CCD8, D14, and D3, respectively, and these gene families have similar motifs and amino acid sequences (Fig. 1-3);. D27, CCD7, and CCD8 are essential for SL biosynthesis, and D14 and D3 for SL recognition and signal transduction [8, 9]; therefore, *FveD27*, *FveCCD7*, and *FveCCD8* likely participate in SL biosynthesis, and *FveD14* and *FveD3* in SL signaling. *Arabidopsis* has one *MAX1* gene (*At2g26170*), which participates in the production of SL precursors [23], whereas rice has five (*Os01g0700900*, *Os01g0701400*, *Os01g0701500*, *Os02g0221900*, and *Os06g0565100*) [24]. Interestingly, Os01g0701500, Os02g0221900, and Os06g0565100 are not involved in the biosynthesis of SL precursors despite having motifs similar to those of *Arabidopsis* MAX1. By contrast, Os01g0700900 and Os01g0701400, which lack four motifs present in *Arabidopsis* MAX1 (Fig. 1b), catalyze the biosynthesis of active SLs [24]. The two FveMAX1 proteins (FveMAX1A and FveMAX1B) of woodland strawberry have the same motif composition as *Arabidopsis* MAX1, but the N-terminal amino acid sequence of FveMAX1B is of unknown function (Fig. 1b). Thus, FveMAX1A, similar to *Arabidopsis* MAX1, likely catalyzes the production of SL precursors, while the function of FvMAX1B is unclear. Further studies should evaluate the functions of FveMAX1A and FveMAX1B in SL biosynthesis. In addition, *Arabidopsis*, rice, and woodland strawberry have one *LBO* gene with the same motif composition (Fig. 1c); *Arabidopsis* LBO acts in the final stages of SL biosynthesis to produce active SLs [10]. Based on our speculation that FveMAX1A has similar activity to *Arabidopsis* MAX1, we postulate that FveLBO has a similar function to *Arabidopsis* LBO in the final stages of SL biosynthesis. Two rice proteins (OsD53 and LOC_Os12g01360), three *Arabidopsis* proteins (AtSMXL6, AtSMXL7, and AtSMXL8) and two woodland strawberry proteins (mrna07912 and mrna20979) clustered in the D53 family of SL signaling proteins (Fig. 3b). OsD53, AtSMXL6, AtSMXL7, and AtSMXL8 can interact with D14 and form complexes with D3/MAX2, resulting in ubiquitination and degradation of OsD53, AtSMXL6, AtSMXL7, and AtSMXL8 in an SL-responsive manner [32–34]. However, the function of rice LOC_Os12g0136, which has high homology to OsD53, is unknown [32]. Woodland strawberry mrna07912 clustered with AtSMXL6 and AtSMXL7, while mrna20979 clustered with AtSMXL8. These proteins have similar motif compositions (Fig. 3**b**), so whether mrna07912 and mrna2097 have similar functions as the other D53 proteins is unclear.

### Expression patterns of SL biosynthetic and signaling genes in vegetative organs of woodland strawberry

SLs are synthesized in plant roots, and can be transported to shoots via the transpiration stream, as suggested by the wild-type (WT) branching pattern of SL biosynthetic mutant scions grafted onto WT rootstocks [11, 47]. In addition, plant shoots may also produce SLs, based on the WT branching phenotypes generated by grafting of WT scions onto rootstocks of *Arabidopsis* and pea SL biosynthetic mutants [15, 23, 48]. In *Arabidopsis*, the expression of the SL biosynthetic genes *MAX3/CCD7* and *MAX4*/*CCD8* was lower and higher, respectively, in root compared with shoot. By contrast, the *MAX1* transcript levels were similar in root and shoot. Notably, the expression of *D27* was extremely low in root [11]. In rice, the expression of *D27*, *CCD7/HTD1*, and *CCD8/D10* is lower in root than in stem [12, 14, 19]. However, this does not mean that localized high concentrations of SLs in certain cell types in the root do not exit, especially some biosynthetic genes, such as *D27*, *CCD7/HTD1*, *CCD8/D10, MAX1*, and *MAX4*/*CCD8* predominantly expressed in root vascular tissue [12, 14, 19, 23, 49]. In fact, natural SLs have been isolated from the roots of diverse plant species [7], while low concentrations of SLs were detected in sorghum shoots [50]. Therefore, we speculate that SLs are produced in root and shoot of woodland strawberry because of the expression of SL biosynthetic genes in root and shoot (Fig. 4a-f), but the expression pattern provides no guidance on where the SL concentrations are highest. *D27*, *CCD7/HTD1* and *CCD8/D10,* are also expressed in rice leaf [12, 14, 19], as are *CCD7* and *CCD8* in tomato leaf [17, 22]. In our study, expression of *D27* and *CCD7*, but not *CCD8*, was detected in leaf and petiole of woodland strawberry (Fig. 4a-c), possibly because *CCD8* is not expressed in these tissues or is expressed at a level below the limit of detection. If the former is true, no CL is produced because D27, MAX3, and MAX4 act on non-mobile substrates in the plastid [11, 47]. However, *FveMAX1A, FveMAX1B* and *FveLBO*, which act downstream of *CCD8*, were expressed in leaf and petiole (Fig. 4d-f), so that SLs may be produced in these locations using CL transported from other tissues.

AtD14/OsD14 is the only SL receptor investigated to date [27–29], and MAX2/D3 is essential for ubiquitin-mediated protein degradation [30, 31]; thus, AtD14/OsD14 and MAX2/D3 are essential for the roles of SLs in the development of different tissues. Consistent with the roles of SLs in plant root and shoot branching, the expression of *AtD14/OsD14* and *MAX2/D3* is similar in the shoot/stem and root of *Arabidopsis* and rice [28, 30, 41, 51]. In woodland strawberry, the expression of *FveD14* and *FveD3* is similar in root and stem (Fig. 4g, h). D53 represses SL signaling in rice tillers and *Arabidopsis* branching, and its expression is relatively high in the shoot base of rice seedlings and axillary buds [32, 33], as well as in the stem of woodland strawberry (Fig. I, J). Because the runners and crowns of strawberry differentiate from stem axillary buds [52], the high expression levels of *FveD53A* and *FveD53B* suggest that these two genes play roles in the development of runners and crowns. In addition, the expression of *FveD14*, *FveD3*, and *FveD53s* was high in leaf and moderate in petiole of woodland strawberry (Fig. 4g-j). The high expression of these genes in leaf is also seen in *Arabidopsis*, pea, petunia, and rice [15, 20, 28, 30, 32, 33, 41, 51]. In *Arabidopsis*, SLs promote the expansion of leaf blade and petiole, producing more open rosettes [9], and regulate leaf senescence in concert with ethylene [53]. In Medicago, SLs increase leaf serration but the overall leaf shape was not affected [54]. In addition, there is a cross-talk between SL and ABA in integrating stress signals to regulate leaf stomatal development and function [36]. However, The effects of SLs on leaf development are complex and deserves in-depth thought and research.

### Possible role for SL in the reproductive growth of woodland strawberry

Floral development is important in plant reproductive growth and is regulated by the plant hormones auxin, GAs, CKs, ethylene, and ABA [55]. For example, auxin not only determines the initiation of flower primordia but also specifies the number of floral organs and pattern formation within a floral organ [56, 57]; CKs positively regulate ovule formation and pistil development [58], and CK response factors integrate the auxin and CK pathways for female reproductive organ development [59]; and GAs act positively in the termination of vegetative development but inhibits flower formation [60, 61]. Ethylene senses DNA damage in the female anther primordium and promotes female flower formation in cucumber [62]. Expression of the SL biosynthetic and signaling genes in rice is high in panicle and even higher than root [12, 14, 19, 32, 33, 41, 51], suggesting that SLs participate in panicle development, but no study focus on this so far. In our study, the transcript levels of putative SL biosynthetic and signaling genes were high and low, respectively, in woodland strawberry flower (stages 1–4), perianth (stages 5–6), and receptacle (stages 6–7) (Fig. 5a), suggesting SLs may accumulate, but SL signaling was inactive in these tissues at the early stages. We speculate that SL accumulation plays a role in progression to the next developmental stage, particularly the carpel (stages 7–8) from the receptacle base (stages 6–7), because *FveD14* expression is activated in carpel at stages 7–8 (Fig. 5a). During carpel and anther development, expression of the SL biosynthetic genes *FveD27*, *FveCCD7*, and *FveCCD8* was low or undetectable (Fig. 5a), indicating that CL is not produced in the carpel and anther. However, the high expression of *FveMAX1s* and *FveLBO* in these tissues suggests synthesis of SLs. The expression of SL signaling genes was higher in carpel than in anther, and the expression of *FveD14* increased with carpel development (Fig. 5a), suggesting SLs play a role in carpel and pistil development. In addition, the expression of biosynthetic and signaling genes was all extremely low in mature pollen, we speculate that SLs are not active in mature pollen. However, expression of *FveD14* was high in the styles (including stigmas) of just-opened flowers but decreased after pollination (Fig. 5a), so fertilization may be required for the functions of SLs in the female organs of woodland strawberry.

### SLs may play a role in the early development of woodland strawberry fruits

Fruit set, development, and ripening are associated with the activities of various plant hormones. Fruit set is defined as the activation of a developmental program that converts the ovary into a developing fruit. Auxin, GAs, and CKs are involved in fruit set [2, 3, 63]. After tomato fertilization, the levels of auxin and CKs dramatically increase to a peak at 2 DPA, and auxin activates GA biosynthesis [64]. In this study, the expression of SL biosynthetic genes decreased upon fertilization both in the achene and receptacle (Fig. 5b), suggesting a decrease in the SL levels in these tissues during fertilization. However, expression of the SL receptor gene *FveD14* decreased in the ovule and carpel/ovary wall of achene, but increased in the receptacle cortex and pitch, upon fertilization (Fig. 5b). Thus, SL signaling may be activated in receptacle, but inhibited in achene by fertilization. This suggests that SLs play different roles in achene and receptacle during fertilization.

In tomato, auxin, CKs, and GAs cooperate to regulate the early fruit development. Auxin, CKs and GAs all have high levels in this process. A summary of the idea was proposed that auxin, CKs and GAs impact each other to promote cell division and expansion, and determine fruit size [65–67]. ABA, BR, and ethylene also play roles in the early development of fruits [2, 3, 64]. In strawberry, the levels of indole-3-acetic acid (IAA), GA_1_, and castasterone increase early in fruit development and decrease prior to color accumulation [68]. The expression of auxin and GA biosynthetic genes is high in the achene (embryo, ghost, and wall) and low in the receptacle (pith and cortex), suggesting that auxin and GA biosynthesis occurs mainly in the achene [43, 69]. However, auxin receptor genes are expressed in both the achene and receptacle, and the expression of GA receptor genes is higher in the receptacle than in the achene. Therefore, auxin signaling is active in the achene and receptacle, and GA signaling in the receptacle. Based on this, the following model has been proposed: auxin is synthesized and stimulates GA biosynthesis in the seed, and auxin and GAs are transported to the receptacle to promote receptacle growth [43]. Consistent with this, removal of achenes inhibits receptacle growth, and removal of some achenes results in abnormally shaped fruits because only the parts of the receptacle adjacent to the remaining achenes continue to grow [70]. Exogenous application of GA_3_, 1-naphthaleneacetic acid (NAA), or GA_3_ plus NAA to non-pollinated fruits induces receptacle enlargement [43]. Therefore, auxin and GAs produced in seeds play important roles in fruit development, but the roles of other hormones in strawberry fruit development are unclear. In our study, the expression of SL biosynthetic and signaling genes was higher in receptacle, particularly in developing pitch, than in achene (Fig. 5b), so SLs may play important roles in receptacle development. However, unlike auxin and GAs, SLs may be synthesized and accumulate in the receptacle to promote its development. Interestingly, in achene, the expression of SL biosynthetic and signaling genes was high in the walls of developing carpel and ovary, ovule (stages 1–2), and embryo (stage 4), but low in ghost (stages 3–5) and embryo (stages 3 and 5) (Fig. 5b). Thus, the roles of SLs differ among tissues and developmental stages.

The ripening of fleshy fruits is an important developmental process in which plant hormones play important roles. Ethylene is the major regulator of most aspects of fruit ripening in climacteric fruits, such as tomato, although auxin, ABA, and GAs are also involved [1–3]. In non-climacteric fruits, such as strawberry, ABA is the major regulator of fruit ripening [68, 71]. Besides, auxin and brassinosteroid also have high levels, and the expression of some biosynthetic and signaling genes increase at the late stages of receptacle ripening [69, 72]. However, exogenous applications of NAA delayed receptacle ripening [73], while exogenous application of BR promote ripening [72]. Ethylene is considered not to be related to strawberry ripening, but influences seed maturation and metabolism [73, 74]. The expression of some JA signaling-related genes decreases from the fruit development to ripening stages, suggesting JA plays a role in early strawberry fruit development, which also correlates negatively with the beginning of the ripening process [75]. *SlCCD7* expression was highest in tomato fruits at the immature green stage [17]. Similarly, in woodland strawberry, expression of the SL biosynthetic and signaling genes was high in the early stages of receptacle development and decreased significantly in ripening receptacle, which was consistent with the change of SL concentrations (Fig. 6). Therefore, we speculate that SLs participate in the regulation of early development, but not ripening, of woodland strawberry fruits. However, understanding of the regulatory mechanisms of SLs in fruit development is still limited. SLs can interact with auxin, CKs and GAs in regulating root development, shoot branching and seed germination [76], together with auxin, CKs, GAs and SLs all have high levels in the early developing fruit, and auxin, CKs and GAs can cooperate to regulate the early fruit development. Therefore, we speculate that SLs regulate the early fruit development through interacting with other hormones, such as auxin, CKs and GAs.

## Conclusions

In conclusion, we identified one *D27*, two *MAX1*, and one *LBO* gene in the SL biosynthetic pathway, and one *D14*, one *D3*, and two *D53* genes in the SL signaling pathway in woodland strawberry. The expression levels of the SL biosynthetic and signaling genes differed among plant tissues and developmental stages. The expression of the SL biosynthetic and signaling genes was high in early receptacle and decreased during receptacle development, suggesting that SLs play a role in woodland strawberry fruit development, especially at the early stages. Woodland strawberry is a useful model plant for studying the molecular and developmental biology of non-climacteric fruits, and our findings provide insight into the function of SLs in fruit development.

## Methods

### Identification of genes and phylogenetic analyses

Protein sequences and annotation information of two monocots (*Oryza sativa* and *Zea mays*) and four eudicots (*Fragaria vesca*, *Arabidopsis thaliana*, *Prunus persica*, and *Malus domestica*) were downloaded from Phytozom (https://phytozome.jgi.doe.gov/pz/portal.html) (Additional file 10)*.* SL-related genes were identified based on review articles (Additional file 11) [8, 9]. The longest sequence was selected while there was redundancy because of alternative splicing. Protein domains were validated using SMART (http://smart.embl-heidelberg.de/), Pfam (http://pfam.xfam.org/search#tabview=tab1), and CDD (https://www.ncbi.nlm.nih.gov/Structure/cdd/wrpsb.cgi). Phylogenetic trees were visualized in FigTree v. 1.3.1 software.

The D27 protein family has no domain annotation on the InterPro website and is classified in the ‘domain of unknown function’ family (Pfam: DUF4033) [11]. To identify D27 homologs in woodland strawberry, a BLASTp search (E-value < 10^− 4^) was performed in the protein databases of six species using the OsD27 and AtD27 sequences as queries in BioEdit. The sequences were filtered to remove redundancy, and the presence of a DUF4033 domain was verified using SMART, Pfam, and CDD. Finally, the sequences of 21 proteins (including OsD27 and AtD27) were obtained. The proteins were aligned in ClustalX 2.1 and exported as FASTA and PHYLIP files. The best JTT + I + G model was identified by Model-Generator [77]. Subsequently, PHYLIP files were used to construct maximum likelihood phylogenetic trees in PhyML with 100 replicates [78].

Using the AtMAX1, PhMAX1, Os01g0700900, Os01g0701400, Os01g0701500, Os02g0221900, and Os06g0565100 sequences as queries, we performed a local BLASTp (E-value < 10^− 25^; other parameters were at their default values) search of the protein databases of six species in BioEdit. Redundant sequences and those that lacked a P450 domain (Pfam: P450) were removed by validation using SMART, Pfam, and CDD, yielding approximately 200 protein sequences. The proteins were aligned in ClustalX 2.1 (using the default parameters) and exported as FASTA and PHYLIP files. The best JTT + G + F model was identified by Model-Generator. The PHYLIP files were used to generate maximum likelihood phylogenetic trees in PhyML with 100 replicates.

To identify LBO homologs in *Oryza sativa*, *Arabidopsis thaliana*, *Zea mays, Fragaria vesca*, *Prunus persica* and *Malus domestica*, the amino acid sequences of *Arabidopsis* LBO protein was used as query to performed a local BLASTp (E-value < 10^− 50^) search in BioEdit. After removing redundancy, 2OG-FeII_Oxy domain were identified using the SMART, Pfam and CDD databases. Subsequently, all the obtained amino acid sequences were multiply aligned by ClustalX 2.1(using default parameters), and the optimum model JTT + I + G was determined by Model-Generator. Finally, a phylogenetic tree was constructed using PhyML with 100 replicates.

Using three OsD14, AtD14, and PhDAD2 as queries, a local BLASTp (E-value < 10^− 4^) search for homologs in the protein databases of six species was performed in BioEdit. After removing redundancy, the remaining sequences were submitted to SMART, Pfam, and CDD to verify the presence of an α/β hydrolyzyme domain. Next, FASTA and PHYLIP files of the 58 identified proteins were obtained by alignment in ClustalX 2.1 with the default parameters. The best WAG+I + G + F model was identified by Model-Generator. A maximum likelihood tree was constructed in PhyML using the default settings and 100 bootstrap replicates.

To identify D3 homologs, we performed a local BLASTp (E-value < 10^− 4^) search in BioEdit software using OsD3, AtMAX2, PsRMS4 and PhMAX2A, PhMAX2B as the query. After removing redundancy, LRR domains were identified using the SMART, Pfam, and CDD databases. In total, 153 F-box LRR proteins (including OsD3, AtMAX2, PsRMS4, PhMAX2A, and PhMAX2B) were aligned using ClustalX 2.1 with the default parameters, and the optimum JTT + G + F model was determined by Model-Generator. A phylogenetic tree was constructed using PhyML. The phylogenetic tree was reconstructed based on four selected groups (bootstrap value > 90) of F-box LRR_7 related proteins [40].

We identified D53 homologs by Local BLASTp (E-Value < 10^− 4^) in BioEdit using rice D53 protein as query. After removing redundancy which did not belong to the Clp (Caseinolytic peptidase) proteins validated by SMART, Pfam and CDD, 84 proteins were multiply aligned by ClustalX 2.1 (using default parameters) for FASTA and PHYLIP format files. The optimum JTT + G + F model was identified by Model-Generator. Finally, PHYLIP format file was used to construct a phylogenetic tree by PhyML with 100 replicates.

### Conserved motif and multiple sequence alignment analysis

Conserved motifs were identified by submitting the complete amino acid sequences to the MEME website (Version 4.10.2, http://meme-suite.org/− tools/meme) [79]; up to 15 motifs were permitted and other parameters were at the default settings. Multiple sequence alignments were performed using ClustalW with the default parameters in BioEdit software. Seven SDPs were decided [28], and putative hydrolase catalytic triad residues were identified [11].

### Plant materials

Seeds of Ruegen (*Fragaria vesca*) were from a seven-generation inbred line provided by Janet Slovin. Woodland strawberry seedlings were grown in 10 × 10 cm pots in a climate room, under a 16 h light (22 °C)/8 h dark (20 °C) cycle with 65% relative humidity. The flowering time was tagged, and fruit samples were collected at the following six developmental stages: SG (about 8 days DPA), LG (about 15 DPA), SW (white pulp with green seeds, about 20 DPA), LW (white pulp with red seeds, about 24 DPA), P (slightly pink pulp with red seeds, about 26 DPA), and R (pulp all red, about 28 DPA) [38, 45, 46]. After removing seeds, the receptacle samples were stored at − 80 °C.

Ruegen seeds were sterilized in 75% alcohol for 5 min followed by 2% (*w*/*v*) sodium hypochlorite for 7 min and washed several times in sterile water. The aseptic seedlings were grown in a climate chamber under a 16 h light (22 °C)/8 h dark (20 °C) cycle with 55 μmol m^− 2^ s^− 1^ irradiance provided by white fluorescent lamps. Murashige and Skoog medium (2% [w/v] sucrose, 7.2 g/L agar) was used for growth of the seedlings. Following growth for up to 40 days, the root (entire root tissue), stem (dwarf stem, entire tissue after removing the petiole and hypocotyl), leaf (all leaves except cotyledons), and petiole were collected. Three biological replicates were performed, each comprising eight seedlings.

### RNA extraction and qPCR analysis

The root, stem, leaf, petiole and receptacle samples stored at − 80 °C were used for total RNA extraction with a modified CTAB method [80].The cDNA was generated by Primerscript RT reagent Kit with gDNA Erase (Takara) according to the manufacturer’s protocol. Quantitative PCR (qPCR) was performed using SYBR Premix Ex Tag (Takara), with *GAPDH* as an internal reference gene [81]. Results were analyzed using the 2^-ΔCT^ method [81]. Three biological and three technical replicates were performed and analyzed. Primers used in this study are listed in Additional file 12.

### Expression analysis

The transcriptome data for different development stages of woodland strawberry were downloaded from (http://bioinformatics.towson.edu/strawberry/) [43]. RPKM values of related genes were transformed in log2 level, and a heatmap was shown using MeV4.8 software.

### SL analysis

The measurement of strigol levels was carried out by high performance liquid chromatography coupled with electrospray ionization multi-tandem mass spectrometry (HPLC-ESI-MSn) at Beijing Forestry University [82–84]. ^2^H_3_-GR24 was synthesized according to Guo et al. (2015) [85].

#### Plant materials and purification procedure

Extraction and prepurification of endogenous SLs from strawberry receptacles were performed according to Liu et al. (2013) [82] with some modifications. Strawberry receptacles were ground to powders using liquid nitrogen, and dehydrated by freeze-drying before hormone analyses. 100 mg dry weight sample was extracted overnight at 4 °C in 6 ml 80% methanol containing 100 ng ^2^H_3_-GR24 as the internal standard. After centrifugation, the supernatant was collected and the residue was re-extracted for 60 min as above. The combined supernatant was loaded onto the Sep-Pak Plus C18 cartridge, and washed with 2 mL 5% methanol, and eluted with 2 mL 80% methanol. The elution was evaporated to aqueous phase in a vacuum at 4 °C, and then loaded onto the MAX cartridge. After being washed with 1 M FA, 0.1 M NH4OH, and 0.1MNH4OH in 60%MeOH, the MAX cartridge was eluted with 1.25 M FA in 70%MeOH. The obtained elution was dried under vacuum and redissolved in 50 μL of 50% methanol in 0.001% formic acid. After filtering, 10 μL of the solution was injected into the HPLC-ESI-MSn system for strigol analysis according to the following optimum procedure. Each sample was analyzed in triplicate.

#### Analysis procedure of strigol

Before analyzing strigol levels of plant samples, the standard sample of strigol purchased from OLChemim Ltd. (Olomouc, Czech Republic) was used to optimize parameters to obtain maximal signal intensities of strigol in HPLC-ESI-MSn. The optimum procedure was as follows: The elution gradient of HPLC was performed with 95% A (0.1% formic acid in water) and 5% B (methanol) at constant flow rate of 0.15 mL/min for 1 min; A decreased to 60% and B increased to 40% in 9 min; then A decreased to 0 and B increased to 100% at 20 min, which should hold on for 5 min; after that, A and B reached the original percent in 1 min and held on for 5 min. Then, a LCQ DECA XP MAX ion trap mass spectrometer system (Thermo-Finnigan) coupled with an ESI source was used with the following parameters: positive ionization mode, Capillary temperature 300 °C, sheath gas: nitrogen, 30abi units, Aux gas: nitrogen, 5abi units (ca. 3.33 L/min), Capillary voltage + 4.0 kV, Tube Lens Offset 30 V, Multiple RF Amplifier 400Vp-p, Multiple 1 Offset − 6.80 V, Multiple 2 Offset − 9.50 V, Inter-multiple Lens Voltage − 16.00 V, Entrance Lens -50 V, Trap DC Offset Voltage -10 V, Capillary temperature 280 °C, Collision energy 35 eV.

## Additional files

Additional file 1: Phylogenetic analysis of D27 protein family identified from rice, *Arabidopsis*, apple, peach, maize, and woodland strawberry. The amino acid sequences of D27 from rice and *Arabidopsis* were used as queries. 21 proteins were obtained after removing redundancy, and the Phylogenetic tree was construct in PhyML with 100 replicates. (JPG 214 kb)

Additional file 2: Distribution of conserved motifs in different protein families. Motif analysis was performed online by MEME, (Version 4.10.2, http://meme-suite.org/− tools/meme) [79]; up to 15 motifs were permitted and other parameters were at the default settings. (DOCX 36 kb)

Additional file 3: Protein sequences of conserved motifs in different protein families. (DOCX 22 kb)

Additional file 4: Phylogenetic analysis of CYP450 protein family identified from rice, *Arabidopsis*, apple, peach, maize, and woodland strawberry. AtMAX1, PhMAX1, Os01g0700900, Os01g0701400, Os01g0701500, Os02g0221900, and Os06g0565100 sequences were used as queries, approximately 200 protein sequences were obtained after removing redundancy, and the Phylogenetic tree was construct in PhyML with 100 replicates. (JPG 1427 kb)

Additional file 5: Phylogenetic analysis of LBO protein family identified from rice, *Arabidopsis*, apple, peach, maize, and woodland strawberry. The amino acid sequences of LBO from *Arabidopsis* were used as queries, approximately 200 protein sequences were obtained after removing redundancy, and the Phylogenetic tree was construct in PhyML with 100 replicates. (JPG 1751 kb)

Additional file 6: Conserved motif analysis of D14 protein families from rice, *Arabidopsis*, apple, peach, maize, petunia, and woodland strawberry. Motifs were identified using MEME software, up to 15 motifs were permitted and other parameters were at the default settings. The 15 motifs were indicated by boxed of different color. Gray lines represent non-conserved sequences. Distribution and protein sequences of conserved motifs were presented in Additional files 2 and 3. (JPG 879 kb)

Additional file 7: Phylogenetic analysis of F-box LRR protein family identified from rice, *Arabidopsis*, apple, peach, maize, and woodland strawberry. The amino acid sequences of OsD3, AtMAX2, PsRMS4 and PhMAX2A, PhMAX2B were used as queries, 153 F-box LRR protein sequences were obtained after removing redundancy, and the Phylogenetic tree was construct in PhyML with 100 replicates. (JPG 1504 kb)

Additional file 8: Phylogenetic analysis of D53 protein family identified from rice, *Arabidopsis*, apple, peach, maize, and woodland strawberry. The amino acid sequences of D53 from rice were used as queries, 84 proteins were obtained after removing redundancy, and the Phylogenetic tree was construct in PhyML with 100 replicates. (JPG 1211 kb)

Additional file 9: The RPKM value of SL biosynthetic and signaling genes in the flower and early-stage fruit of woodland strawberry. The transcriptome data was downloaded from (http://bioinformatics.towson.edu/strawberry/) [43]. (DOCX 21 kb)

Additional file 10: Databases for all six species we used in this research. (DOCX 12 kb)

Additional file 11: The functionally analyzed proteins of plant species used in this study. (DOCX 13 kb)

Additional file 12: Primers used in qPCR analysis of this study. (DOCX 13 kb)

## Abbreviations

ABA: Abscisic acid
BR: Brassinolide
CCD7: Carotenoid Cleavage Dioxygenase 7
CCD8: Carotenoid Cleavage Dioxygenase 8
CKs: Cytokinins
CL: Carlactone
CLA: Carlactonoic acid
D27: DWARF27
GAs: Gibberellins
IAA: Indole-3-acetic acid
IPA1: Ideal Plant Architecture1
JA: Jasmonic acid
LBO: Oxidoreductase-like enzyme LATERAL BRANCHING OXIDOREDUCTASE
MAX1: Cytochrome P450 monooxygenase MORE AXILLARY GROWTH 1
MeCLA: Methyl carlactonoate
NAA: 1-naphthaleneacetic acid
SA: Salicylic acid
SLs: Strigolactones
TPR2: TOPLESS (TPL)-related protein 2
