# Supplementary figures and images for: Identification and expression analysis of strigolactone biosynthetic and signaling genes reveal strigolactones are involved in fruit development of the woodland strawberry (Fragaria vesca)
Source: BMC Plant Biol. 2019 Feb 14;19:73. doi: 10.1186/s12870-019-1673-6 (PMC6376702; doi:10.1186/s12870-019-1673-6)

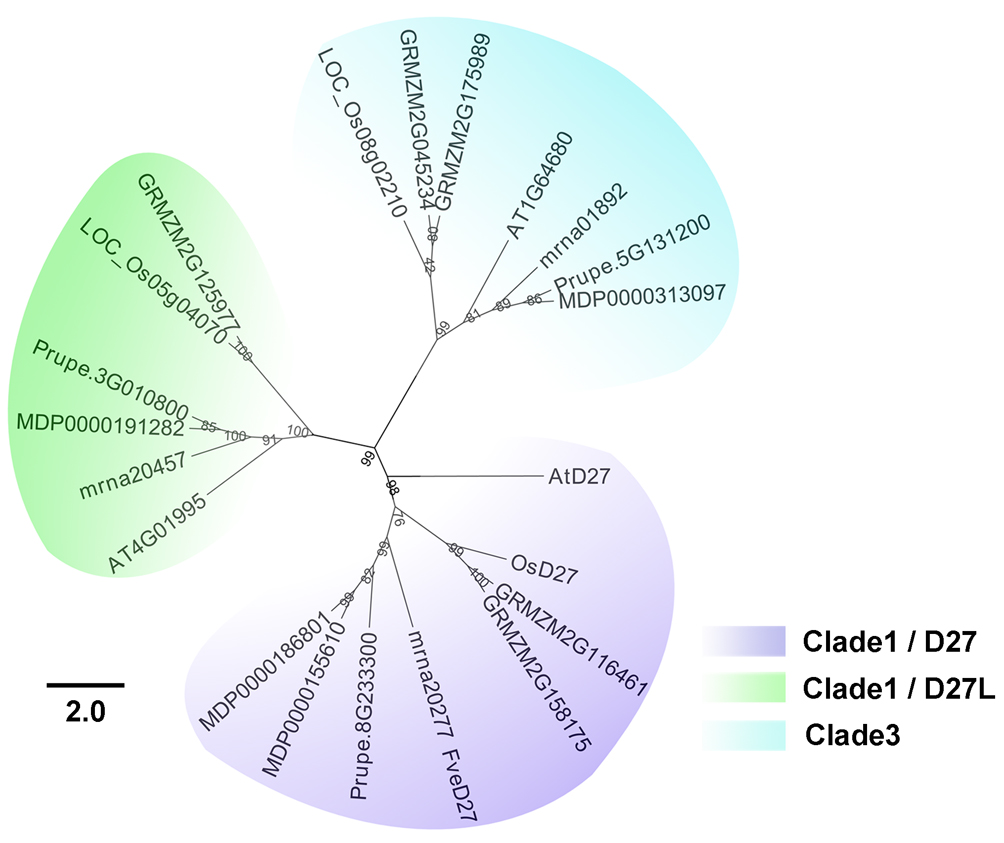

Supplement: Supplementary file 1 — Phylogenetic analysis of D27 protein family identified from rice, Arabidopsis, apple, peach, maize, and woodland strawberry. The amino acid sequences of D27 from rice and Arabidopsis were used as queries. 21 proteins were obtained after removing redundancy, and the Phylogenetic tree was construct in PhyML with 100 replicates. (JPG 214 kb) [file 12870_2019_1673_MOESM1_ESM.jpg]

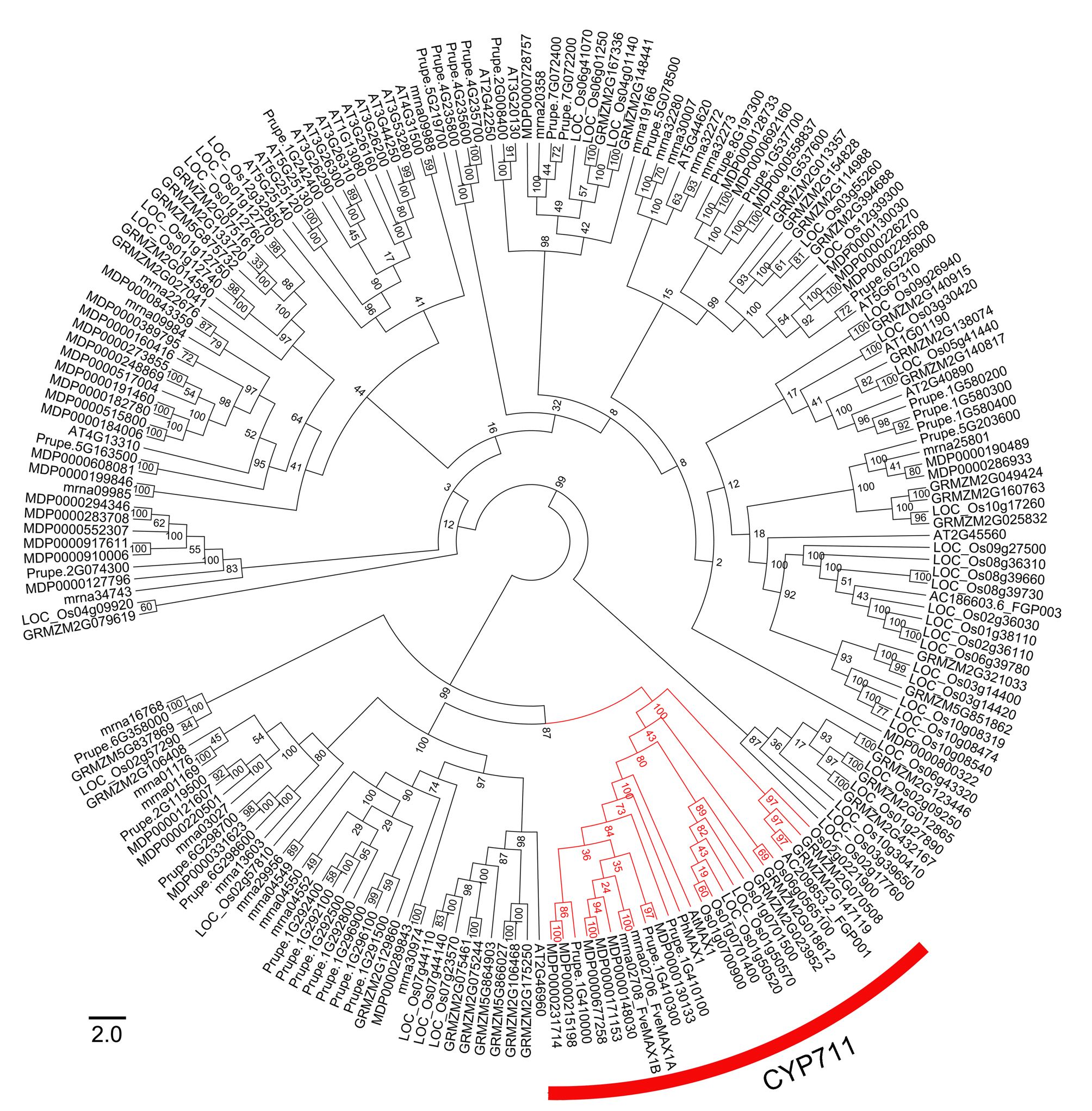

Supplement: Supplementary file 4 — Phylogenetic analysis of CYP450 protein family identified from rice, Arabidopsis, apple, peach, maize, and woodland strawberry. AtMAX1, PhMAX1, Os01g0700900, Os01g0701400, Os01g0701500, Os02g0221900, and Os06g0565100 sequences were used as queries, approximately 200 protein sequences were obtained after removing redundancy, and the Phylogenetic tree was construct in PhyML with 100 replicates. (JPG 1427 kb) [file 12870_2019_1673_MOESM4_ESM.jpg]

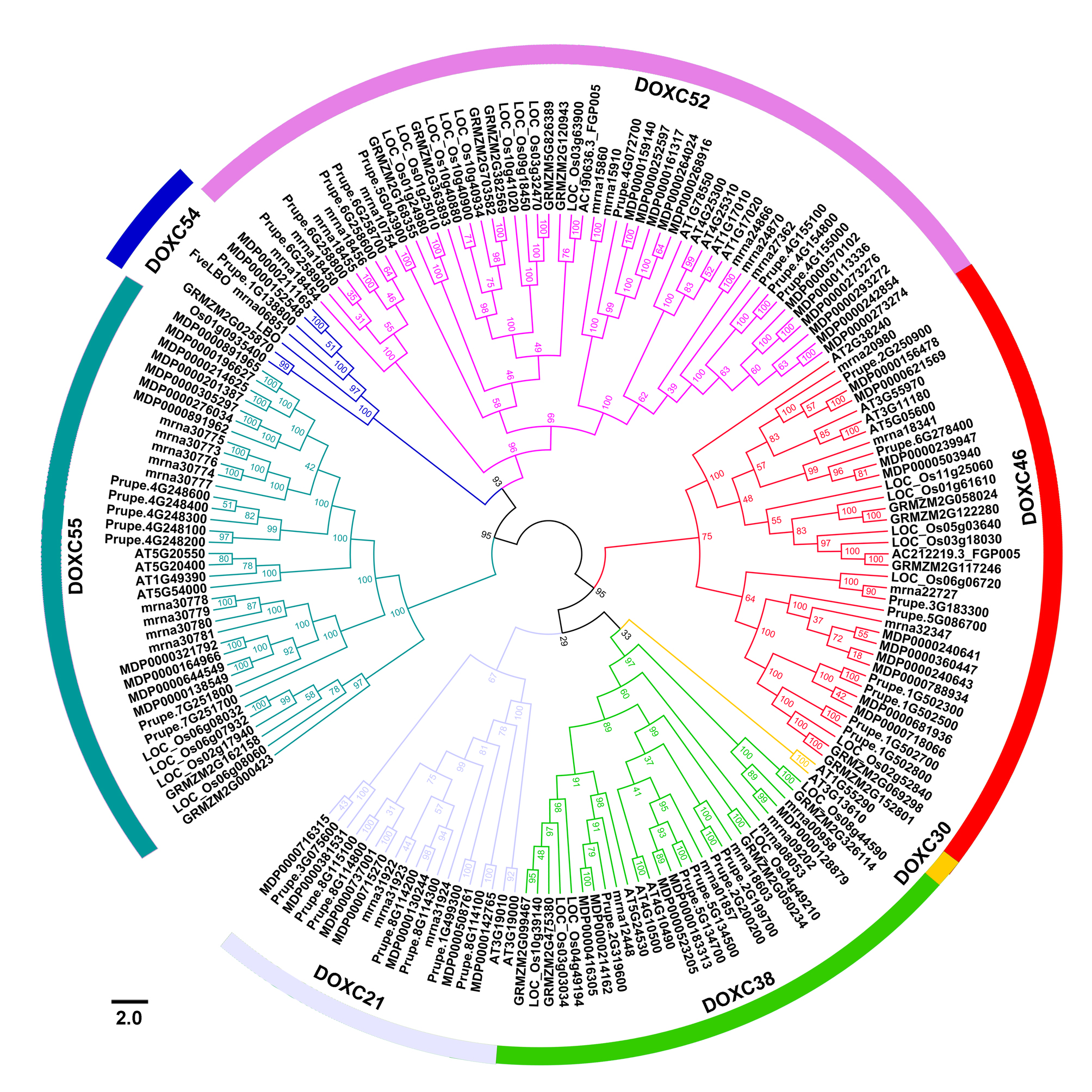

Supplement: Supplementary file 5 — Phylogenetic analysis of LBO protein family identified from rice, Arabidopsis, apple, peach, maize, and woodland strawberry. The amino acid sequences of LBO from Arabidopsis were used as queries, approximately 200 protein sequences were obtained after removing redundancy, and the Phylogenetic tree was construct in PhyML with 100 replicates. (JPG 1751 kb) [file 12870_2019_1673_MOESM5_ESM.jpg]

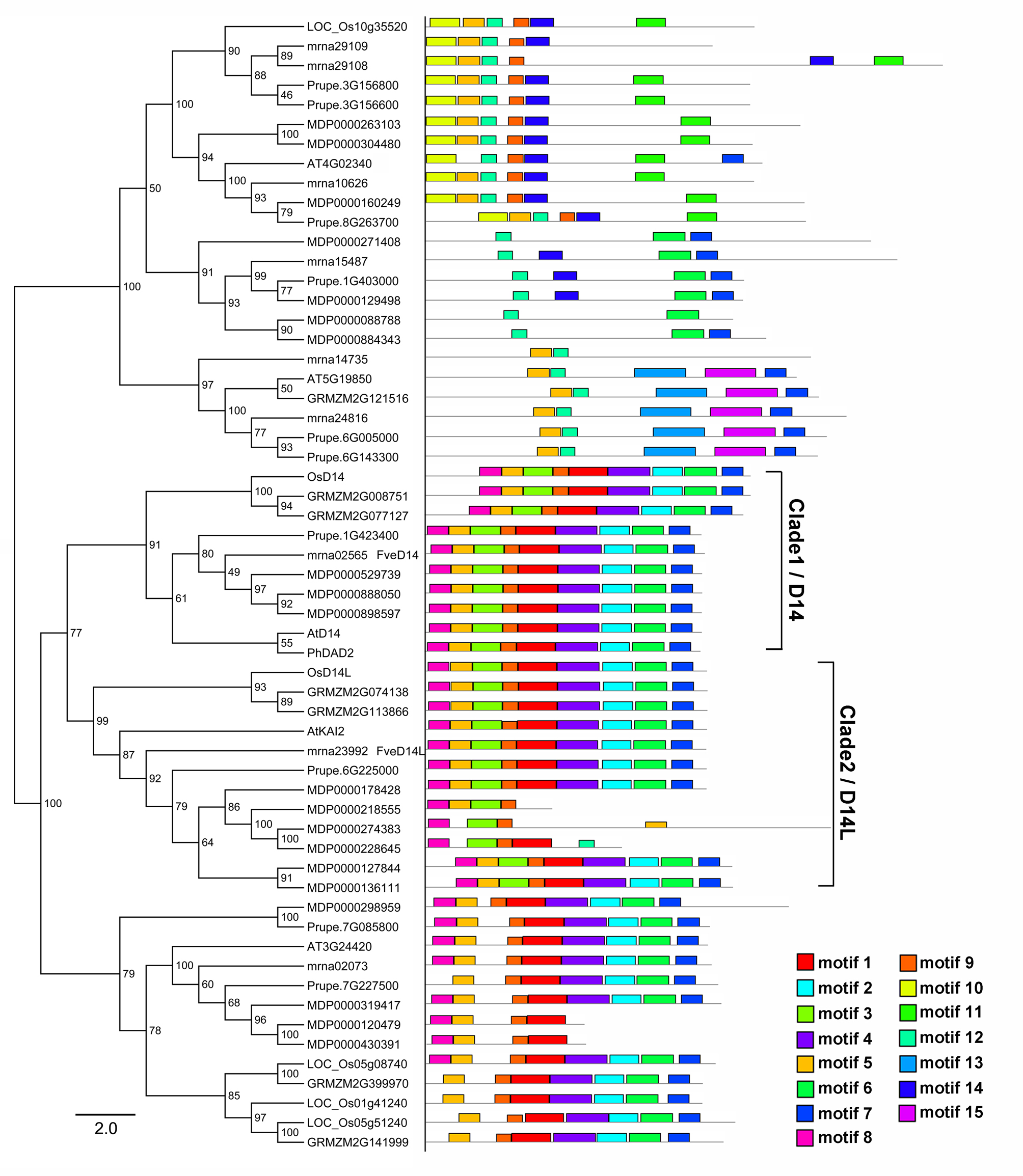

Supplement: Supplementary file 6 — Conserved motif analysis of D14 protein families from rice, Arabidopsis, apple, peach, maize, petunia, and woodland strawberry. Motifs were identified using MEME software, up to 15 motifs were permitted and other parameters were at the default settings. The 15 motifs were indicated by boxed of different color. Gray lines represent non-conserved sequences. Distribution and protein sequences of conserved motifs were presented in Additional files 2 and 3. (JPG 879 kb) [file 12870_2019_1673_MOESM6_ESM.jpg]

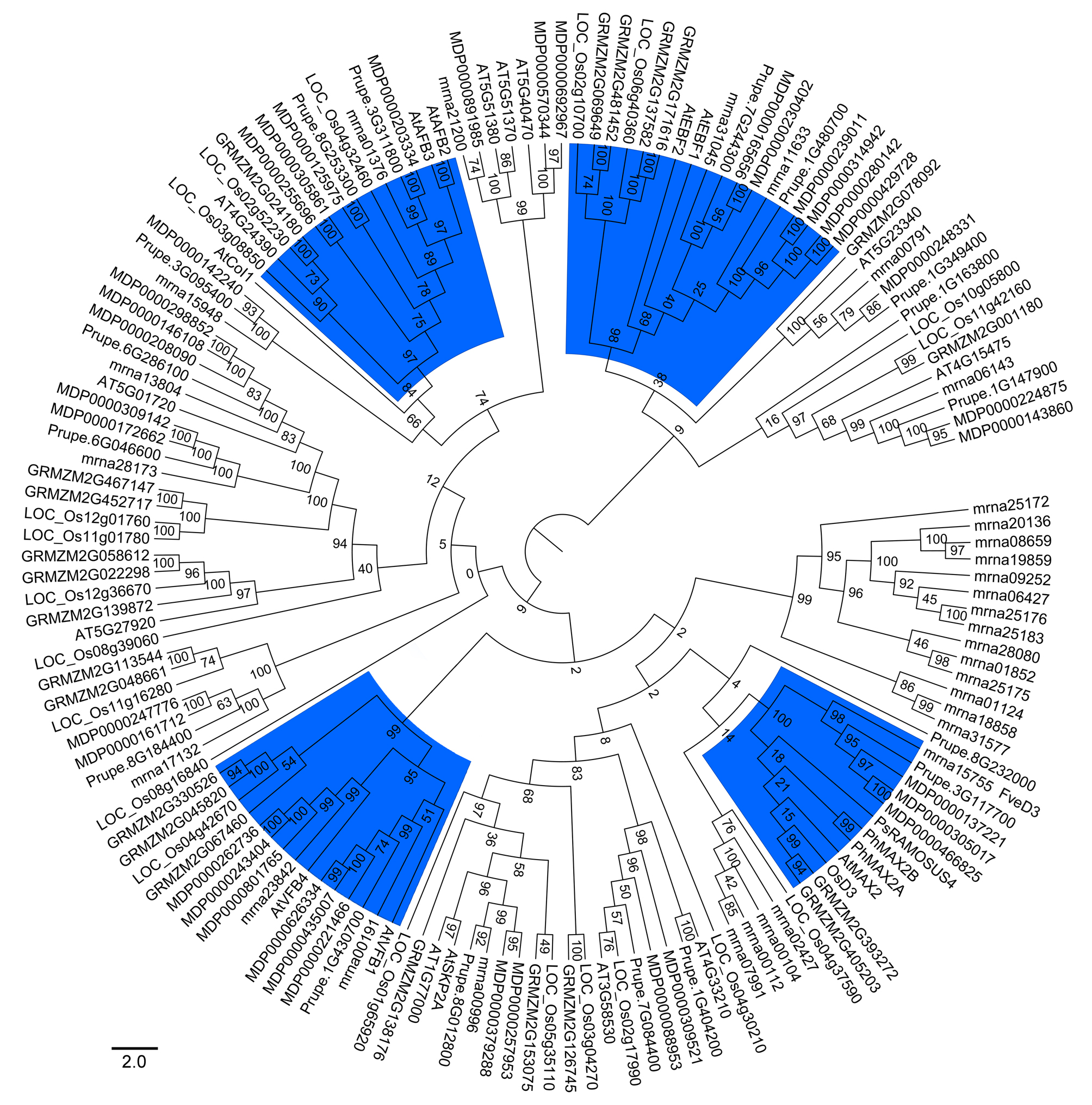

Supplement: Supplementary file 7 — Phylogenetic analysis of F-box LRR protein family identified from rice, Arabidopsis, apple, peach, maize, and woodland strawberry. The amino acid sequences of OsD3, AtMAX2, PsRMS4 and PhMAX2A, PhMAX2B were used as queries, 153 F-box LRR protein sequences were obtained after removing redundancy, and the Phylogenetic tree was construct in PhyML with 100 replicates. (JPG 1504 kb) [file 12870_2019_1673_MOESM7_ESM.jpg]

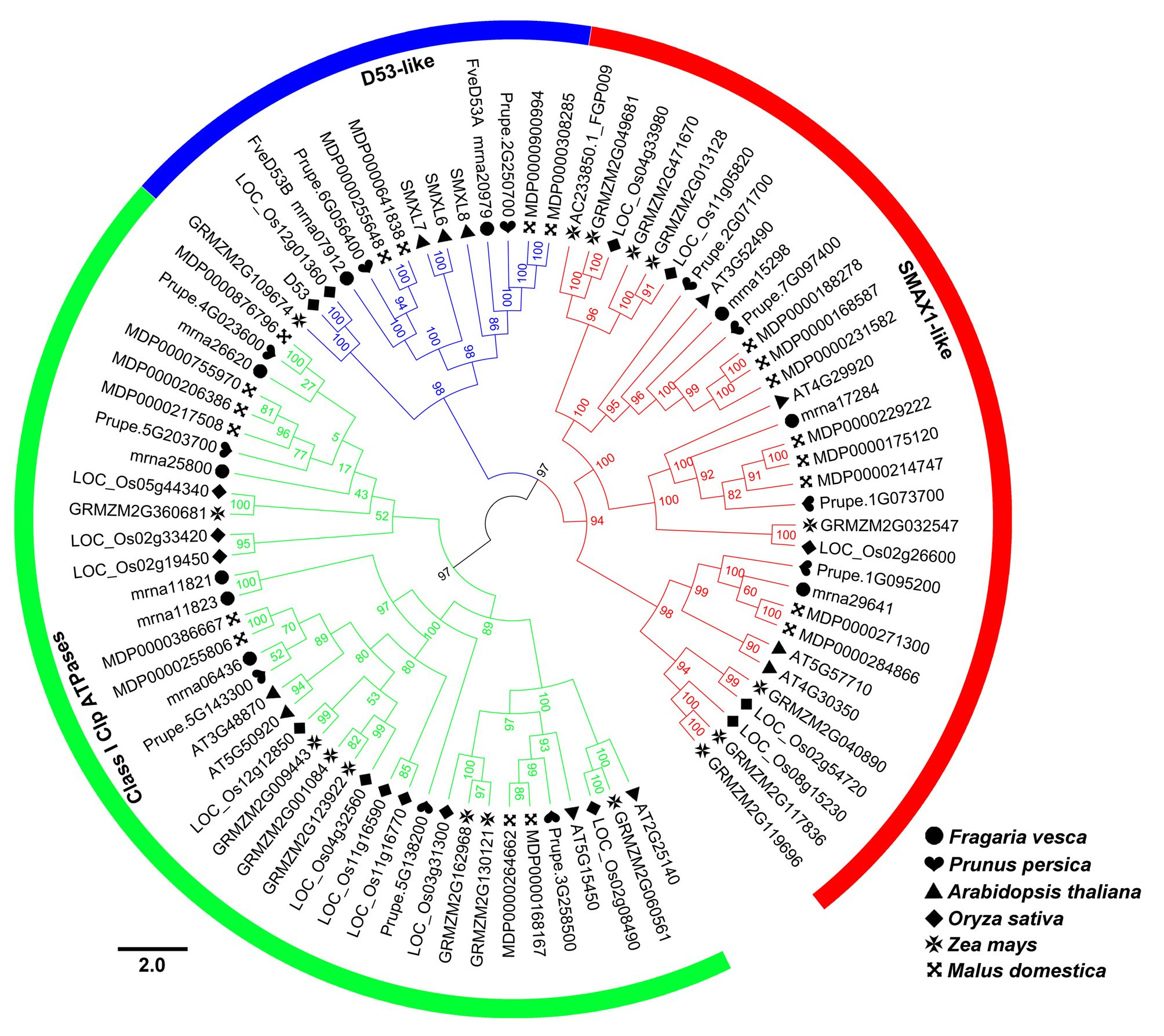

Supplement: Supplementary file 8 — Phylogenetic analysis of D53 protein family identified from rice, Arabidopsis, apple, peach, maize, and woodland strawberry. The amino acid sequences of D53 from rice were used as queries, 84 proteins were obtained after removing redundancy, and the Phylogenetic tree was construct in PhyML with 100 replicates. (JPG 1211 kb) [file 12870_2019_1673_MOESM8_ESM.jpg]
