# Supplementary material for: Identification and expression analysis of strigolactone biosynthetic and signaling genes reveal strigolactones are involved in fruit development of the woodland strawberry (Fragaria vesca)
Source: BMC Plant Biol. 2019 Feb 14;19:73. doi: 10.1186/s12870-019-1673-6 (PMC6376702; doi:10.1186/s12870-019-1673-6)
Supplement: Supplementary file 2 — Distribution of conserved motifs in different protein families. Motif analysis was performed online by MEME, (Version 4.10.2, http://meme-suite.org/− tools/meme) [79]; up to 15 motifs were permitted and other parameters were at the default settings. (DOCX 36 kb) [file 12870_2019_1673_MOESM2_ESM.docx]

**Additional file 2:** Distribution of conserved motifs in different protein families. Motif analysis was performed online by MEME, (Version 4.10.2, http://meme-suite.org/- tools/meme) [78]; up to 15 motifs were permitted and other parameters were at the default settings.

| **Subfamilies** | | **Proteins** | **Motifs** | | | | | | | | | | | | | | | | |  |
| --- | --- | --- | --- | --- | --- | --- | --- | --- | --- | --- | --- | --- | --- | --- | --- | --- | --- | --- | --- | --- |
|  |  |  | **1** | **2** | **3** | **4** | **5** | **6** | **7** | **8** | **9** | **10** | **11** | | **12** | **13** | **14** | | **15** |  |
| **D27** | | AtD27 | 1 | 1 |  | 1 | 1 |  |  |  |  |  |  | | 1 |  |  | | 1 |  |
|  |  | OsD27 | 1 | 1 | 1 | 1 | 1 |  |  |  |  | 1 |  | |  | 1 | 1 | |  |  |
|  |  | FveD27 | 1 | 1 | 1 | 1 | 1 | 1 | 1 | 1 |  |  | 1 | |  |  |  | |  |  |
|  |  | Prupe.8G233300 | 1 | 1 | 1 | 1 | 1 | 1 | 1 | 1 |  |  |  | |  |  |  | |  |  |
|  |  | GRMZM2G116461 | 1 | 1 | 1 | 1 | 1 |  |  |  | 1 | 1 |  | |  |  |  | | 1 |  |
|  |  | GRMZM2G158175 | 1 | 1 | 1 | 1 | 1 |  |  |  | 1 | 1 |  | |  |  |  | |  |  |
|  |  | MDP0000155610 | 1 | 1 | 1 | 1 | 1 | 1 |  |  |  |  | 1 | |  | 1 |  | |  |  |
|  |  | MDP0000186801 | 1 | 1 | 1 | 1 | 1 | 1 | 1 | 1 |  |  | 1 | | 1 |  | 1 | |  |  |
| **CYP711** | Os02g0221900 | 1 | 1 | 1 | 1 | 1 | 1 | 1 | 1 | 1 | 1 | 1 | | 1 | 1 |  | |  |  |  |
|  | GRMZM2G070508 | 1 | 1 | 1 | 1 | 1 | 1 | 1 | 1 | 1 | 1 | 1 | | 1 | 1 |  | |  |  |  |
|  | GRMZM2G147119 | 1 |  |  | 1 |  |  | 1 |  |  | 1 | 1 | |  |  |  | |  |  |  |
|  | AC209853.2 | 1 |  |  |  |  |  |  |  |  | 1 | 1 | |  |  |  | |  |  |  |
|  | Os06g0565100 | 1 | 1 | 1 | 1 | 1 | 1 | 1 | 1 | 1 | 1 | 1 | | 1 | 1 |  | |  |  |  |
|  | GRMZM2G018612 | 1 | 1 | 1 | 1 | 1 | 1 | 1 | 1 | 1 | 1 | 1 | | 1 | 1 |  | |  |  |  |
|  | GRMZM2G023952 | 1 | 1 | 1 | 1 | 1 | 1 | 1 | 1 | 1 | 1 | 1 | | 1 | 1 |  | |  |  |  |
|  | Os01g0701500 | 1 | 1 | 1 | 1 |  | 1 | 1 | 1 | 1 | 1 | 1 | | 1 |  |  | |  |  |  |
|  | LOC_Os01g50570 |  |  | 1 |  |  |  |  |  | 1 |  |  | |  |  |  | |  |  |  |
|  | LOC_Os01g50520 |  |  | 1 |  |  |  |  |  | 1 |  |  | |  |  |  | |  |  |  |
|  | Os01g0701400 | 1 | 1 |  | 1 | 1 |  | 1 | 1 |  | 1 | 1 | |  | 1 |  | |  |  |  |
|  | Os01g0700900 | 1 | 1 |  | 1 | 1 |  | 1 | 1 |  | 1 | 1 | | 1 | 1 |  | |  |  |  |
|  | AtMAX1 | 1 | 1 | 1 | 1 | 1 | 1 | 1 | 1 | 1 | 1 | 1 | | 1 | 1 |  | |  |  |  |
|  | PhMAX1 | 1 | 1 | 1 | 1 | 1 | 1 | 1 | 1 | 1 | 1 | 1 | | 1 | 1 | 1 | |  |  |  |
|  | Prupe.1G410100 | 1 | 1 | 1 | 1 | 1 | 1 | 1 | 1 | 1 | 1 | 1 | | 1 | 1 | 1 | |  |  |  |
|  | MDP0000130133 | 1 | 1 | 1 | 1 | 1 | 1 | 1 | 1 | 1 | 1 | 1 | | 1 | 1 | 1 | |  |  |  |
|  | Prupe.1G410300 | 1 | 1 | 1 | 1 | 1 | 1 | 1 | 1 | 1 | 1 | 1 | | 1 | 1 | 1 | | 1 |  |  |
|  | FveMAX1A | 1 | 1 | 1 | 1 | 1 | 1 | 1 | 1 | 1 | 1 | 1 | | 1 | 1 | 1 | | 1 |  |  |
|  | FveMAX1B | 1 | 1 | 1 | 1 | 1 | 1 | 1 | 1 | 1 | 1 | 1 | | 1 | 1 | 1 | | 1 |  |  |
|  | MDP0000148030 | 1 | 1 | 1 | 1 | 1 | 1 | 1 | 1 | 1 | 1 | 1 | | 1 | 1 | 1 | | 1 |  |  |
|  | MDP0000171153 | 1 |  |  |  |  |  | 1 |  |  | 1 | 1 | | 1 |  |  | | 1 |  |  |
|  | MDP0000677258 | 1 |  |  |  |  |  | 1 |  |  | 1 | 1 | | 1 |  |  | | 1 |  |  |
|  | Prupe.1G410000 | 1 | 1 |  | 1 | 1 |  | 1 | 1 |  | 1 | 1 | | 1 | 1 |  | | 1 |  |  |
|  | MDP0000215198 | 1 | 1 | 1 | 1 | 1 | 1 | 1 | 1 | 1 | 1 | 1 | | 1 | 1 | 1 | | 1 |  |  |
|  | MDP0000231714 | 1 | 1 | 1 | 1 | 1 | 1 | 1 | 1 | 1 | 1 | 1 | | 1 | 1 |  | | 1 |  |  |
| **DOXC54** | MDP0000211165 | 1 | 1 | 1 | 1 | 1 | 1 | 1 | 1 | 1 | 1 | 1 | | 1 |  |  | | 1 |  |  |
|  | MDP0000152548 | 1 | 1 | 1 | 1 | 1 | 1 | 1 | 1 | 1 | 1 | 1 | | 1 |  |  | |  |  |  |
| Continued last page | | | | | | | | | | | | | | | | | | |  |  |
| **DOXC54** | Prupe.1G138800 | 1 | 1 | 1 | 1 | 1 | 1 | 1 | 1 | 1 | 1 | 1 | | 1 |  |  |  | |  |  |
|  | FveLBO | 1 | 1 | 1 | 1 | 1 | 1 | 1 | 1 | 1 | 1 | 1 | | 1 |  |  |  | |  |  |
|  | GRMZM2G025870 | 1 | 1 | 1 | 1 | 1 | 1 | 1 | 1 |  |  | 1 | |  | 1 | 1 |  | |  |  |
|  | Os01g0935400 | 1 | 1 | 1 | 1 | 1 |  | 1 | 1 |  |  | 1 | |  | 1 | 1 | 1 | |  |  |
|  | LBO | 1 | 1 | 1 | 1 | 1 | 1 | 1 | 1 | 1 | 1 | 1 | | 1 |  |  |  | |  |  |
| **D14** | | OsD14 | 1 | 1 | 1 | 1 | 1 | 1 | 1 | 1 | 1 |  |  | |  |  |  | |  | |
|  |  | GRMZM2G008751 | 1 | 1 | 1 | 1 | 1 | 1 | 1 | 1 | 1 |  |  | |  |  |  | |  | |
|  |  | GRMZM2G077127 | 1 | 1 | 1 | 1 | 1 | 1 | 1 | 1 | 1 |  |  | |  |  |  | |  | |
|  |  | Prupe.1G423400 | 1 | 1 | 1 | 1 | 1 | 1 | 1 | 1 | 1 |  |  | |  |  |  | |  | |
|  |  | FveD14 | 1 | 1 | 1 | 1 | 1 | 1 | 1 | 1 | 1 |  |  | |  |  |  | |  | |
|  |  | MDP0000529739 | 1 | 1 | 1 | 1 | 1 | 1 | 1 | 1 | 1 |  |  | |  |  |  | |  | |
|  |  | MDP0000888050 | 1 | 1 | 1 | 1 | 1 | 1 | 1 | 1 | 1 |  |  | |  |  |  | |  | |
|  |  | MDP0000898597 | 1 | 1 | 1 | 1 | 1 | 1 | 1 | 1 | 1 |  |  | |  |  |  | |  | |
|  |  | AtD14 | 1 | 1 | 1 | 1 | 1 | 1 | 1 | 1 | 1 |  |  | |  |  |  | |  | |
|  |  | PhDAD2 | 1 | 1 | 1 | 1 | 1 | 1 | 1 | 1 | 1 |  |  | |  |  |  | |  | |
| **D14L** | | OsD14L | 1 | 1 | 1 | 1 | 1 | 1 | 1 | 1 | 1 |  |  | |  |  |  | |  | |
|  |  | GRMZM2G074138 | 1 | 1 | 1 | 1 | 1 | 1 | 1 | 1 | 1 |  |  | |  |  |  | |  | |
|  |  | GRMZM2G113866 | 1 | 1 | 1 | 1 | 1 | 1 | 1 | 1 | 1 |  |  | |  |  |  | |  | |
|  |  | AtKAI2 | 1 | 1 | 1 | 1 | 1 | 1 | 1 | 1 | 1 |  |  | |  |  |  | |  | |
|  |  | FveD14L | 1 | 1 | 1 | 1 | 1 | 1 | 1 | 1 | 1 |  |  | |  |  |  | |  | |
|  |  | Prupe.6G225000 | 1 | 1 | 1 | 1 | 1 | 1 | 1 | 1 | 1 |  |  | |  |  |  | |  | |
|  |  | MDP0000178428 | 1 | 1 | 1 | 1 | 1 | 1 | 1 | 1 | 1 |  |  | |  |  |  | |  | |
|  |  | MDP0000218555 |  |  | 1 |  | 1 |  |  | 1 | 1 |  |  | |  |  |  | |  | |
|  |  | MDP0000274383 |  |  | 1 |  | 1 |  |  | 1 | 1 |  |  | |  |  |  | |  | |
|  |  | MDP0000228645 | 1 |  | 1 |  |  |  |  | 1 | 1 | 1 |  | |  |  |  | |  | |
|  |  | MDP0000127844 | 1 | 1 | 1 | 1 | 1 | 1 | 1 | 1 | 1 |  |  | |  |  |  | |  | |
|  |  | MDP0000136111 | 1 | 1 | 1 | 1 | 1 | 1 | 1 | 1 | 1 |  |  | |  |  |  | |  | |
| **D3/MAX2** | | PhMAX2B | 1 | 1 |  | 1 |  | 1 | 1 | 1 |  | 1 | 1 | | 1 |  | 1 | |  | |
|  |  | PhMAX2A | 1 | 1 |  | 1 |  | 1 | 1 | 1 |  | 1 | 1 | | 1 |  | 1 | |  | |
|  |  | AtMAX2 | 1 | 1 |  | 1 |  | 1 | 1 | 1 |  | 1 | 1 | | 1 |  | 1 | |  | |
|  |  | PsRAMOSUS4 | 1 | 1 |  | 1 |  | 1 | 1 | 1 |  | 1 | 1 | | 1 |  | 1 | |  | |
|  |  | FveD3_mrna15755 | 1 | 1 |  | 1 |  | 1 | 1 | 1 |  | 1 | 1 | | 1 |  | 1 | |  | |
|  |  | Prupe.3G117700 | 1 | 1 |  | 1 |  | 1 | 1 | 1 |  | 1 | 1 | | 1 |  | 1 | |  | |
|  |  | MDP0000137221 | 1 | 1 |  | 1 |  | 1 | 1 | 1 |  | 1 | 1 | | 1 |  | 1 | |  | |
|  |  | MDP0000305017 | 1 | 1 |  | 1 |  | 1 | 1 | 1 |  | 1 | 1 | | 1 |  | 1 | |  | |
|  |  | MDP0000466825 | 1 | 1 |  | 1 |  | 1 | 1 | 1 |  | 1 | 1 | | 1 |  | 1 | |  | |
|  |  | OsD3 | 1 | 1 |  | 1 |  | 1 | 1 | 1 |  | 1 |  | | 1 |  | 1 | |  | |
|  |  | GRMZM2G393272 | 1 | 1 |  |  |  |  | 1 | 1 |  | 1 |  | |  |  | 1 | |  | |
|  |  | GRMZM2G405203 | 1 | 1 |  | 1 |  | 1 | 1 | 1 |  | 1 |  | | 1 |  | 1 | |  | |
| Continued last page | | | | | | | | | | | | | | | | | | | | |
| **AFB** | | LOC_Os03g08850 | 1 | 1 |  |  | 1 | 1 | 1 | 1 | 1 | 1 |  | |  |  |  | |  | |
|  |  | MDP0000255696 | 1 | 1 |  |  | 1 | 1 |  | 1 | 1 | 1 |  | |  |  |  | |  | |
|  |  | MDP0000305861 | 1 | 1 |  |  | 1 | 1 | 1 | 1 | 1 | 1 | 1 | |  |  |  | |  | |
|  |  | MDP0000125975 | 1 | 1 |  |  | 1 | 1 | 1 | 1 | 1 | 1 | 1 | |  |  |  | |  | |
|  |  | Prupe.8G253300 | 1 | 1 |  |  | 1 | 1 | 1 | 1 | 1 | 1 | 1 | |  |  |  | |  | |
|  |  | LOC_Os04g32460 | 1 | 1 |  |  | 1 | 1 | 1 | 1 | 1 | 1 |  | |  |  |  | |  | |
|  |  | mrna01376 | 1 | 1 |  |  | 1 | 1 | 1 | 1 | 1 | 1 |  | |  |  |  | |  | |
|  |  | Prupe.3G311800 | 1 | 1 |  |  | 1 | 1 | 1 | 1 | 1 | 1 |  | |  |  |  | |  | |
|  |  | MDP0000203334 | 1 | 1 |  |  |  | 1 | 1 | 1 | 1 | 1 | 1 | |  |  |  | |  | |
|  |  | AtAFB3 | 1 | 1 |  |  | 1 | 1 | 1 | 1 | 1 | 1 |  | |  |  |  | |  | |
|  |  | AtAFB2 | 1 | 1 |  |  | 1 | 1 | 1 | 1 | 1 | 1 |  | |  |  |  | |  | |
|  |  | AT4G24390 | 1 | 1 |  |  | 1 | 1 | 1 | 1 | 1 | 1 |  | |  |  |  | |  | |
|  |  | LOC_Os02g52230 | 1 | 1 |  |  | 1 | 1 | 1 | 1 | 1 | 1 |  | |  |  |  | |  | |
|  |  | GRMZM2G024180 | 1 | 1 |  |  | 1 | 1 | 1 | 1 | 1 | 1 |  | |  |  |  | |  | |
| **VFB** | | AtVFB4 | 1 | 1 |  |  | 1 | 1 | 1 | 1 |  | 1 | 1 | |  | 1 |  | |  | |
|  |  | mrna23842 | 1 | 1 |  |  | 1 | 1 | 1 | 1 |  | 1 | 1 | |  | 1 |  | |  | |
|  |  | MDP0000801765 | 1 | 1 |  |  | 1 | 1 | 1 | 1 |  | 1 | 1 | |  | 1 |  | |  | |
|  |  | MDP0000262736 | 1 | 1 |  |  | 1 | 1 | 1 | 1 |  | 1 | 1 | |  | 1 |  | |  | |
|  |  | MDP0000243404 | 1 | 1 |  |  | 1 | 1 | 1 | 1 |  | 1 | 1 | |  | 1 |  | |  | |
|  |  | mrna00191 | 1 | 1 |  |  | 1 | 1 | 1 | 1 |  | 1 | 1 | |  | 1 |  | |  | |
|  |  | Prupe.1G430700 | 1 | 1 |  |  | 1 | 1 | 1 | 1 |  | 1 | 1 | |  | 1 |  | |  | |
|  |  | MDP0000221466 | 1 | 1 |  |  | 1 | 1 | 1 | 1 |  | 1 | 1 | |  | 1 |  | |  | |
|  |  | AtVFB1 | 1 | 1 |  |  | 1 | 1 | 1 | 1 |  | 1 | 1 | |  | 1 |  | |  | |
|  |  | GRMZM2G067460 | 1 | 1 |  |  | 1 | 1 | 1 | 1 |  | 1 | 1 | |  | 1 |  | |  | |
|  |  | LOC_Os04g42670 | 1 | 1 |  |  | 1 | 1 | 1 | 1 |  | 1 | 1 | |  | 1 |  | |  | |
|  |  | GRMZM2G045820 | 1 | 1 |  |  | 1 | 1 | 1 | 1 |  | 1 | 1 | |  | 1 |  | |  | |
|  |  | GRMZM2G330526 | 1 | 1 |  |  | 1 | 1 | 1 | 1 |  | 1 | 1 | |  | 1 |  | |  | |
| **EBF** | | mrna31045 | 1 | 1 | 1 |  | 1 | 1 | 1 | 1 |  | 1 | 1 | |  | 1 |  | | 1 | |
|  |  | Prupe.7G244300 | 1 | 1 | 1 |  | 1 | 1 | 1 | 1 |  | 1 | 1 | |  | 1 |  | | 1 | |
|  |  | MDP0000230402 | 1 | 1 | 1 |  | 1 | 1 | 1 | 1 |  | 1 | 1 | |  | 1 |  | | 1 | |
|  |  | MDP0000165656 | 1 | 1 | 1 |  | 1 | 1 | 1 | 1 |  | 1 | 1 | |  | 1 |  | | 1 | |
|  |  | mrna11633 | 1 | 1 | 1 |  | 1 | 1 | 1 | 1 |  | 1 | 1 | |  | 1 |  | | 1 | |
|  |  | Prupe.1G480700 | 1 | 1 | 1 |  | 1 | 1 | 1 | 1 |  | 1 | 1 | |  | 1 |  | | 1 | |
|  |  | MDP0000314942 | 1 | 1 | 1 |  | 1 | 1 | 1 | 1 |  | 1 | 1 | |  | 1 |  | | 1 | |
|  |  | MDP0000239011 | 1 | 1 | 1 |  | 1 | 1 | 1 | 1 |  | 1 | 1 | |  | 1 |  | | 1 | |
|  |  | MDP0000429728 | 1 | 1 | 1 |  | 1 | 1 | 1 | 1 |  | 1 | 1 | |  | 1 |  | | 1 | |
|  |  | MDP0000280142 | 1 | 1 | 1 |  | 1 | 1 | 1 | 1 |  | 1 | 1 | |  | 1 |  | | 1 | |
|  |  | AtEBF1 | 1 | 1 | 1 |  | 1 | 1 | 1 | 1 |  | 1 | 1 | |  | 1 |  | | 1 | |
|  |  | AtEBF2 | 1 | 1 | 1 |  | 1 | 1 | 1 | 1 |  | 1 | 1 | |  |  |  | | 1 | |
| Continued last page | | | | | | | | | | | | | | | | | | | | |
| **EBF** | | LOC_Os06g40360 | 1 | 1 | 1 |  | 1 | 1 | 1 | 1 |  | 1 | 1 | |  | 1 |  | | 1 | |
|  |  | GRMZM2G137582 | 1 | 1 | 1 |  | 1 | 1 | 1 | 1 |  | 1 | 1 | |  | 1 |  | | 1 | |
|  |  | GRMZM2G171616 | 1 | 1 | 1 |  | 1 | 1 | 1 | 1 |  | 1 | 1 | |  | 1 |  | | 1 | |
|  |  | LOC_Os02g10700 | 1 | 1 | 1 |  | 1 | 1 | 1 | 1 |  | 1 | 1 | |  | 1 |  | | 1 | |
|  |  | GRMZM2G481452 | 1 | 1 | 1 |  | 1 | 1 | 1 | 1 |  | 1 | 1 | |  | 1 |  | | 1 | |
|  |  | GRMZM2G069649 | 1 | 1 | 1 |  | 1 | 1 | 1 | 1 |  | 1 | 1 | |  | 1 |  | | 1 | |
| **D53-like** | | GRMZM2G109674 | 1 | 1 | 1 | 1 | 1 | 1 |  | 1 | 1 | 1 | 1 | 1 | |  | 1 | | 1 | |
|  |  | D53 | 1 | 1 | 1 | 1 | 1 | 1 | 1 | 1 | 1 | 1 | 1 | 1 | |  | 1 | | 1 | |
|  |  | LOC_Os12g01360 | 1 | 1 | 1 | 1 | 1 | 1 | 1 | 1 | 1 | 1 | 1 | 1 | |  | 1 | | 1 | |
|  |  | FveD53B | 1 | 1 | 1 | 1 | 1 | 1 | 1 | 1 | 1 | 1 | 1 | 1 | | 1 | 1 | | 1 | |
|  |  | FveD53A |  | 1 | 1 | 1 | 1 | 1 | 1 | 1 | 1 | 1 | 1 | 1 | |  | 1 | | 1 | |
|  |  | Prupe.6G056400 | 1 | 1 | 1 | 1 | 1 | 1 | 1 | 1 | 1 | 1 | 1 | 1 | | 1 | 1 | | 1 | |
|  |  | MDP0000255648 | 1 | 1 | 1 | 1 | 1 | 1 | 1 | 1 | 1 | 1 | 1 | 1 | | 1 | 1 | | 1 | |
|  |  | MDP0000641838 | 1 | 1 | 1 | 1 | 1 | 1 | 1 | 1 | 1 | 1 | 1 | 1 | | 1 | 1 | | 1 | |
|  |  | SMXL7 | 1 | 1 | 1 | 1 | 1 | 1 | 1 | 1 | 1 | 1 | 1 | 1 | | 1 | 1 | | 1 | |
|  |  | SMXL6 | 1 | 1 | 1 | 1 | 1 | 1 | 1 | 1 | 1 | 1 | 1 | 1 | |  | 1 | | 1 | |
|  |  | SMXL8 | 1 | 1 | 1 | 1 | 1 | 1 | 1 | 1 | 1 |  | 1 |  | |  |  | | 1 | |
|  |  | Prupe.2G250700 | 1 | 1 | 1 | 1 | 1 | 1 | 1 | 1 | 1 | 1 | 1 | 1 | | 1 | 1 | | 1 | |
|  |  | MDP0000900994 | 1 | 1 | 1 | 1 | 1 | 1 | 1 | 1 | 1 | 1 | 1 | 1 | | 1 | 1 | | 1 | |
|  |  | MDP0000308285 | 1 | 1 | 1 | 1 | 1 | 1 | 1 | 1 | 1 | 1 | 1 | 1 | | 1 | 1 | | 1 | |
