# Supplementary material for: Identification and expression analysis of strigolactone biosynthetic and signaling genes reveal strigolactones are involved in fruit development of the woodland strawberry (Fragaria vesca)
Source: BMC Plant Biol. 2019 Feb 14;19:73. doi: 10.1186/s12870-019-1673-6 (PMC6376702; doi:10.1186/s12870-019-1673-6)
Supplement: Supplementary file 3 — Protein sequences of conserved motifs in different protein families. (DOCX 22 kb) [file 12870_2019_1673_MOESM3_ESM.docx]

**Additional file 3:** Protein sequences of conserved motifs in different protein families

| Proteins | Motifs | E-value | Motif content |
| --- | --- | --- | --- |
| D27 family  (Figure 1A) | motif 1 | 6.3e-256 | YFAAFTTIFFAWLVGPCEVRESEVBGRKEKNVVYIPKCRFLEETNCVGMC |
|  | motif 2 | 6.8e-184 | NMCKIPSQSFIKDSLGTPVYMVPNFDDMSCEMIFGQQPPEI |
|  | motif 3 | 2.7e-166 | YRDNWFDKLAIGHLSQNVQAASGLKNNKSGYESLIEAATAASRKFNPDKQ |
|  | motif 4 | 3.3e-071 | VLQALDRAFPKPILSLJKVILPPSKFARE |
|  | motif 5 | 4.0e-054 | DPALKQPCFKLCKVAKQNH |
|  | motif 6 | 5.5e-021 | KLRRCSIVAVLTTPAENINIATDKR |
|  | motif 7 | 8.3e-008 | TLAHPRHVHEH |
|  | motif 8 | 5.9e-006 | MEASHFLQSR |
|  | motif 9 | 3.3e-002 | MARPHQVPPPATAT |
|  | motif 10 | 1.3e+000 | QVNCSI |
|  | motif 11 | 2.0e+002 | PADAAVEGKRV |
|  | motif 12 | 4.2e+002 | INYLMKN |
|  | motif 13 | 2.7e+003 | MARPRE |
|  | motif 14 | 3.4e+003 | RLYVLR |
|  | motif 15 | 3.5e+003 | DTKQQH |
| CYP711 family  (Figure 1B) | motif 1 | 6.3e-789 | TPDYISALTYEHLLAGSATTAFTLSSVVYLVAGHPEVEKKLLAEIDGFGP |
|  | motif 2 | 1.9e-695 | PKGTWVWLALGVLAKDPKNFPEPEKFRPERFDPNGEEEKQRHPYAFIPFG |
|  | motif 3 | 2.5e-671 | YGPDVFSVLAKQYGPIFRFHMGRQPLVIVADAELCREVGIKKFKDIPNRS |
|  | motif 4 | 2.8e-604 | EFINZHIYSTTQLKMDLSGSLSIILGLLVPILQEPFRQLLKRIPGTMDWK |
|  | motif 5 | 2.3e-586 | GPRACIGQKFALQEIKLSLIHLYRKYVFRHSPNMESPLELEFGIVLNFKN |
|  | motif 6 | 3.1e-480 | SPLHQKGLFFTRDARWSTMRNTILSLYQPSHLASLVPTMQSFIESATQNL |
|  | motif 7 | 4.5e-415 | KEZEDITFSBLSLRLATDVIGQAAFGVBFGLSKPQSISDSI |
|  | motif 8 | 2.2e-330 | QVIKEAMRFYMVSPLVARETSRZVEIGGY |
|  | motif 9 | 7.7e-297 | YLYAPYWGVRRVPGPPTJPLVGHLPLLAK |
|  | motif 10 | 2.4e-189 | VERTNRKLSGRLDEIVAKRMKDSE |
|  | motif 11 | 4.2e-171 | KDFLSLILNARESEGVSKNVF |
|  | motif 12 | 4.1e-146 | DQMPTAHDLQHKFPY |
|  | motif 13 | 1.5e-037 | VKLRVIKR |
|  | motif 14 | 6.1e-025 | TNVSLVSTIFTVLAILAGVLG |
|  | motif 15 | 4.0e-020 | IDGKDNNDDDV |
| DOXC54 family  (Figure 1C) | motif 1 | 1.9e-215 | FFMLPLEEKKKYPMAPGTVQGYGQAFVLSEDQKLDWCNMFALGVEPNFIR |
|  | motif 2 | 7.1e-199 | LGLKGDVFEKMFGEAVQAVRMNYYPPCSRPDLVLGLSPHSDGSALTVLQQ |
|  | motif 3 | 3.1e-146 | VLTNGKYKSVEHRAVTHKEKDRLSIVTFYAPSYEVELGPMPEL |
|  | motif 4 | 4.5e-118 | CKYRRYNHGEYSKHYVTNKLQGKKTLEFA |
|  | motif 5 | 3.5e-087 | ACEEWGFFQVVNHGIDLSJLESIEKVAKE |
|  | motif 6 | 1.8e-081 | MAPIPISPIKVGHIDDVQELRKSKPSIIPERFVRDMTERPT |
|  | motif 7 | 3.1e-065 | DDRWVPVKPIPNALVINIGDT |
|  | motif 8 | 9.2e-067 | PEKFSETVEVYSKEVRKLCQNLLKYIAMS |
|  | motif 9 | 8.6e-036 | MPCSSDIPTINFSKLSKGTTDEJKSEISQ |
| Continued Table S1 | | | |
| DOXC54 family (Figure 1C) | motif 10 | 1.2e-012 | KGNSVGLQILK |
|  | motif 11 | 5.6e-012 | NPMLWPT |
|  | motif 12 | 1.4e+000 | VDENNP |
|  | motif 13 | 1.1e+002 | DDECAQERIPVIDVGELQR |
|  | motif 14 | 1.0e+003 | DAGCAGLQV |
|  | motif 15 | 2.4e+003 | QRRLCSATIDNCRYIRDG |
| α/β-Hydrolase family  (Additional file 6) | motif 1 | 3.4e-846 | CVFVGHSVSAMIGCJASIRRPDLFSKLVLIGASPRYLN |
|  | motif 2 | 3.2e-473 | VQEFSRTLFNMRPDIALSVARTVFNSDLR |
|  | motif 3 | 3.8e-440 | RVILYDLMGAGSTNPDYFDFRRYSTLEGY |
|  | motif 4 | 2.9e-640 | DVDYHGGFEQEEJEQLFSAIESNYKAWVSGFAPLAVGADVP |
|  | motif 5 | 7.9e-468 | HGFGTDQSVWRHLLPYLAQDY |
|  | motif 6 | 2.8e-447 | LGLVRVPCHIVQTAKDVAVPVSVAEYLKKHL |
|  | motif 7 | 1.5e-313 | VEVLPTEGHLPQLSAPDLLNP |
|  | motif 8 | 1.3e-226 | IVEEALNVRVVGSGQQVLVLA |
|  | motif 9 | 4.3e-191 | ADDLJAJLDELGVKS |
|  | motif 10 | 7.0e-112 | MEKIEHTTVQTNGIKLHVAEIGSGPKVLL |
|  | motif 11 | 6.9e-114 | PPWLSEEDLNVYASKFEKSGFTGPLNYYR |
|  | motif 12 | 1.0e-110 | VYAPDLRGYGDSDKP |
|  | motif 13 | 1.3e-130 | MLHIKKQPWYGRPLIKSFQNLLRNTDVGKYFFKTVATPESVRNILCQCYH |
|  | motif 14 | 2.3e-102 | FLVGHDWGAVVAYWVALFRPDRV |
|  | motif 15 | 2.1e-124 | EPGAVDVFLEFICYSGGPLPEELLPQVKCPVLIAWGEKDPWEPIELGRAY |
| F-box LRR family  (Figure 3A) | motif 1 | 3.6e-863 | PNLERLDLCGCPGITDKGLAAIAAKCPNLKKLCJEGC |
|  | motif 2 | 3.8e-622 | WSLDGVGDEGLLAVAKGCPNLQELRLIGV |
|  | motif 3 | 1.2e-560 | KVKLQALNITDVSLAVIGHYGKAVTDLVLTGLPNVSERGFWVMGNAQGLQ |
|  | motif 4 | 1.2e-383 | ELDYWPPQDRDVNQRSLSLPAAGLLQECVTLRKLFIHGTAHEHFMMFLLR |
|  | motif 5 | 7.2e-364 | LRGCREITDAGLAAIAKNC |
|  | motif 6 | 4.9e-337 | RNACSLVCKRWLRLE |
|  | motif 7 | 2.5e-605 | SBEGLZAJGSGCPKLRKLKJKDCPGVGDA |
|  | motif 8 | 6.9e-388 | PGVTBVGLVAIAEGCPKLRKL |
|  | motif 9 | 5.9e-343 | FIGNCYAVSPERVIRRFPGLRSLTLKGKPHFADFNLVPHGWGGY |
|  | motif 10 | 2.0e-327 | PGLAAVLDLCPQLEELSLKRL |
|  | motif 11 | 2.7e-309 | DSIDDLPDECLFEIFRRLGGG |
|  | motif 12 | 5.1e-292 | HKPTZREFGLSCLAGYPKLSKMKLDCGDTVGYALTAPPGQMDLSLWERFF |
|  | motif 13 | 1.7e-338 | LVELDLSKCQVSDFGLAAJSSADQLELLH |
|  | motif 14 | 1.8e-282 | KGLRTLACLLRKTLVDVSISCCKNLDAAASLRALEPIRDRIZRLHIDCVW |
|  | motif 15 | 7.1e-251 | RSLEGKKATDVRLAAIAVGTASRGGLGKL |
| D53-like family  (Figure 3B) | motif 1 | 9.4e-403 | MPTPVSAARQCLTPEAARALDEAVAVARRRSHAQTTSLHAVSALLSLPSS |
|  | motif 2 | 3.4e-334 | KVELKHLILSILDDPVVSRVFAEAGFRSSEIKLAILRPPPT |
|  | motif 3 | 6.1e-330 | ADEPPVSNSLMAAIKRSQANQRRHPESFHLYHQHSQQQSAS |
| Continued Table S1 | | | |
| D53-like family  (Figure 3B) | motif 4 | 5.0e-329 | RGKTVVDYVAGELSRKPHSVVFLENVDKADFLAQSSLSZAIRTGKFRDSH |
|  | motif 5 | 3.1e-269 | HRGKVWLIGATASYETYLKFLSRFPTIEKDWDLQLLPITSS |
|  | motif 6 | 9.9e-270 | RRDIWLNLVGPDRVGKKKIALALAEVLYGSQENLICVDLNSQDRGIDSD |
|  | motif 7 | 1.3e-267 | VVFKPFDFDALAEKIVKEIKNEFKKIFGSECLLEIDSGVMEQJLAAAWLS |
|  | motif 8 | 4.8e-208 | ACARARSSAYSPRLQFKALELCVGVSLDR |
|  | motif 9 | 7.0e-139 | YPKSSLMGSFVPFGGFFSAPS |
|  | motif 10 | 2.3e-195 | DLGGQFDPSDVKSLRRVLTEKVGWQTEAISAISQAIARCRS |
|  | motif 11 | 4.2e-113 | QEESSELSKRANKTSRSFLDLNLPVEETE |
|  | motif 12 | 3.4e-139 | KTKDDGTTLSSKVSALQKKWBDICQRLH |
|  | motif 13 | 2.1e-125 | GREISINNIIFVTTSTIKKGSKSLSSENEPSKYSEERILQAKKCQMQIRN |
|  | motif 14 | 2.2e-110 | YQSFPRCHQCNEKYEQEVASI |
|  | motif 15 | 9.3e-076 | GRNPLLVGVCAAEAL |
