# Supplementary material for: Identification and expression analysis of strigolactone biosynthetic and signaling genes reveal strigolactones are involved in fruit development of the woodland strawberry (Fragaria vesca)
Source: BMC Plant Biol. 2019 Feb 14;19:73. doi: 10.1186/s12870-019-1673-6 (PMC6376702; doi:10.1186/s12870-019-1673-6)
Supplement: Supplementary file 9 — The RPKM value of SL biosynthetic and signaling genes in the flower and early-stage fruit of woodland strawberry. The transcriptome data was downloaded from (http://bioinformatics.towson.edu/strawberry/) [43]. (DOCX 21 kb) [file 12870_2019_1673_MOESM9_ESM.docx]

**Additional file 9:** The RPKM value of SL biosynthetic and signaling genes in the flower and early-stage fruit of woodland strawberry. The transcriptome data was downloaded from (http://bioinformatics.towson.edu/strawberry/ Publication.aspx) [43].

| GeneNo  Sample | FveD27  mrna20277 | FveCCD7  mrna04863 | FveCCD8  mrna08839 | FveMAX1A  mrna02706 | FveMAX1B  mrna02708 | FveLBO  mrna06851 | FveD14  mrna02565 | FveD3  mrna15755 | FveD53A  mrna20979 | FveD53B  mrna07912 |
| --- | --- | --- | --- | --- | --- | --- | --- | --- | --- | --- |
| 1_4_Flowered_B_C | 1.373842737 | 1.605632239 | 4.41564738 | 10.58909413 | 14.57992739 | 103.3215085 | 4.31274141 | 22.5958979 | 13.00128159 | 121.6481136 |
| 5_6_Perianth | 0.326724691 | 1.150836436 | 0.690948332 | 4.805026934 | 6.561865967 | 33.3320629 | 6.075893863 | 22.79975262 | 12.73190265 | 56.74480551 |
| 6-7receptacle | 0.269176225 | 0.77482502 | 1.011566168 | 17.29635001 | 19.36034099 | 31.64806365 | 6.053474207 | 17.56932551 | 11.96264871 | 57.14638093 |
| 10microspores | 1.852005198 | 1.246209105 | 3.796281117 | 1.845016499 | 3.429855296 | 0.850100747 | 1.975472211 | 13.44494381 | 6.589992241 | 7.495332007 |
| Pollen | 0 | 0.674739139 | 0 | 0.104033721 | 0.131720395 | 0.201094402 | 0.411894025 | 1.57871555 | 4.314206446 | 0.103371091 |
| 7-8Anther | 0.093797664 | 0 | 0 | 1.206381469 | 4.068987971 | 14.6711291 | 14.11021861 | 25.64905495 | 17.35878953 | 29.4424148 |
| Anther9 | 1.724012251 | 0.0901792 | 0 | 0.962126885 | 3.526785678 | 30.50167067 | 14.13632938 | 70.63689369 | 13.05559784 | 47.97320906 |
| Anther10 | 0.558302117 | 0.024984347 | 0 | 0.560285803 | 3.746588145 | 35.78317243 | 10.90510441 | 30.5614681 | 16.2472893 | 53.41475918 |
| Anther11 | 0.629074017 | 0.0862443 | 0 | 0.313233165 | 2.418283626 | 55.64177555 | 12.24875982 | 21.6324798 | 14.45392239 | 52.59135739 |
| Anther12 | 0.923775377 | 0.140323126 | 0 | 0.663495612 | 2.332943404 | 109.5806174 | 9.575726586 | 18.90162471 | 14.56421898 | 28.39928519 |
| 7-8Carpel | 0.05373008 | 0 | 0 | 1.716631218 | 4.052050425 | 16.21064101 | 23.82465989 | 55.01941562 | 19.78549448 | 44.67380656 |
| 9carpel | 0.029757347 | 0 | 0 | 0.655480098 | 2.31646746 | 7.415920463 | 41.12282393 | 59.93597217 | 28.32033149 | 62.76349684 |
| 10carpels | 0.05659592 | 0 | 0 | 0.747403356 | 3.015457469 | 11.52268974 | 48.31082795 | 82.5549066 | 16.42994328 | 65.31548065 |
| 10-11carpel | 0.066472049 | 0.931487021 | 0 | 1.449231233 | 4.819290236 | 47.55507187 | 66.28569356 | 99.19805132 | 31.01486077 | 65.43915855 |
| 12carpel | 0.12011006 | 0.592692635 | 1.805497849 | 2.572621522 | 4.444261387 | 109.5700097 | 3.170905597 | 45.2423409 | 27.80084707 | 59.66186627 |
| Style-1 | 0.03734074 | 28.72428996 | 0 | 1.103778771 | 4.125815267 | 51.1846095 | 142.9429984 | 124.7959108 | 18.21907513 | 33.47769515 |
| style2 | 0 | 30.37735307 | 0.023863618 | 1.073274825 | 3.545608258 | 101.5016951 | 44.0646749 | 139.4346424 | 16.57801305 | 64.84062104 |
| Embryo3 | 0 | 0.091054167 | 0 | 0 | 0.934600862 | 0.456941227 | 10.77298463 | 20.01823523 | 16.47128696 | 8.406666469 |
| Embryo4 | 0 | 4.056438 | 1.369908553 | 2.243093542 | 4.475508261 | 1.392753096 | 77.5378673 | 52.18660692 | 50.43114398 | 137.5472076 |
| Embryo5 | 0.106957345 | 0.109250358 | 0 | 0.244020599 | 0.407341472 | 0.72425463 | 1.147029238 | 7.473571686 | 7.310616706 | 3.576532774 |
| Ovule1 | 0.232408512 | 0.057502036 | 0 | 0.700479763 | 3.48362365 | 35.91724592 | 73.85704176 | 38.407477 | 39.51156473 | 63.60459804 |
| Ovule2 | 0.19665893 | 0.368027009 | 0 | 1.177752115 | 3.303581933 | 46.75163054 | 35.62771932 | 45.19734049 | 33.40139047 | 83.41276122 |
| ghost3 | 0.900755838 | 1.591913639 | 0 | 0.479939296 | 2.108021173 | 75.81651686 | 8.044541301 | 27.60690245 | 28.45341342 | 93.99766568 |
| ghost4 | 0.557924865 | 1.711089375 | 0 | 0.585830721 | 2.89492387 | 86.28932046 | 8.221310193 | 28.24961902 | 32.26148171 | 78.84347559 |
| ghost5 | 0.040602863 | 0.784732645 | 0 | 0.421830949 | 2.058619077 | 166.2719792 | 5.236684094 | 24.16175294 | 27.23434459 | 78.88182588 |
| wall1 | 0.04762251 | 0.1471795 | 0 | 2.084785616 | 7.474382789 | 30.88405464 | 117.5817373 | 43.24013405 | 36.68838194 | 53.19331786 |
| wall2 | 0.079184348 | 0.049520342 | 0 | 1.150386584 | 4.142428399 | 40.79950882 | 69.28570873 | 55.63018257 | 39.33262573 | 78.62579744 |
| wall3 | 0.304604169 | 0.024186636 | 0 | 1.223472329 | 3.071149133 | 25.78520999 | 38.94368792 | 45.44342435 | 31.06157453 | 60.71718124 |
| wall4 | 0.214839482 | 0.058447582 | 0 | 1.521281935 | 4.66949572 | 16.90840745 | 38.9939018 | 43.44622445 | 30.15668887 | 52.54363362 |
| Wall5 | 0.099606246 | 0.078899129 | 0 | 2.424056913 | 6.848863432 | 23.71618177 | 41.30890696 | 48.42713351 | 34.28682204 | 67.55316655 |
| cortex1 | 0.098219501 | 6.527363849 | 0 | 13.46282621 | 23.70416272 | 67.55316655 | 12.73982245 | 40.5310034 | 71.18666493 | 220.1192546 |
| Cortex2 | 0.027366821 | 0.566928653 | 0.925090037 | 4.105676332 | 9.477284665 | 416.3940534 | 42.62531792 | 52.11785971 | 67.94668378 | 126.1993014 |
| cortex3 | 0.050389598 | 2.52876601 | 1.02497953 | 1.874371525 | 3.651703117 | 167.7299914 | 29.55323882 | 45.65306489 | 109.9855446 | 67.5331821 |
| cortex4 | 0 | 4.056438 | 1.369908553 | 2.243093542 | 4.475508261 | 90.41815497 | 77.5378673 | 52.18660692 | 137.5472076 | 50.43114398 |
| cortex5 | 0 | 2.69487475 | 1.76196021 | 0.921622989 | 2.103008287 | 31.39639746 | 34.65048718 | 38.80082854 | 137.8912634 | 40.42878153 |
| pitch1 | 0.091815579 | 9.639501818 | 0.04313045 | 89.23465057 | 129.4881849 | 735.7385905 | 10.2299108 | 15.46851218 | 108.9795453 | 150.8056226 |
| pitch2 | 0 | 1.572432 | 1.027122993 | 31.96541871 | 62.64280925 | 285.003158 | 304.306174 | 102.2288799 | 81.51323698 | 130.6659036 |
| pith3 | 0 | 8.442948954 | 9.988732053 | 36.8420297 | 80.90042292 | 148.3941647 | 74.71808283 | 49.73810807 | 187.8420322 | 78.46298386 |
| pith4 | 0 | 12.83727396 | 9.488247385 | 46.3153763 | 107.5816244 | 94.35334327 | 131.6978806 | 52.28993447 | 196.6111141 | 58.06180677 |
| pith5 | 0 | 18.87118573 | 11.82617675 | 65.00052202 | 163.8695731 | 58.06180677 | 91.32514343 | 30.08779108 | 309.7445851 | 66.09527301 |
