# Supplementary material for: Identification and expression analysis of strigolactone biosynthetic and signaling genes reveal strigolactones are involved in fruit development of the woodland strawberry (Fragaria vesca)
Source: BMC Plant Biol. 2019 Feb 14;19:73. doi: 10.1186/s12870-019-1673-6 (PMC6376702; doi:10.1186/s12870-019-1673-6)
Supplement: Supplementary file 10 — Databases for all six species we used in this research. (DOCX 12 kb) [file 12870_2019_1673_MOESM10_ESM.docx]

**Additional file 10:** Databases for all six species we used in this research

| Species | Common name | Version | Resources |
| --- | --- | --- | --- |
| *Arabidopsis thaliana* | *Arabidopsis* | TAIR 10 | Phytozome V11 |
| *Fragaria vesca* | strawberry | Version 1.1 | Phytozome V11 |
| *Oryza sativa* | Rice | Version 7.0 | Phytozome V11 |
| *Prunus persica* | Peach | Version 2.1 | Phytozome V11 |
| *Malus domestica* | Apple | Version 1.0 | Phytozome V11 |
| *Zea mays* | Maize | Version 6a | Phytozome V11 |
