# Supplementary material for: Identification and expression analysis of strigolactone biosynthetic and signaling genes reveal strigolactones are involved in fruit development of the woodland strawberry (Fragaria vesca)
Source: BMC Plant Biol. 2019 Feb 14;19:73. doi: 10.1186/s12870-019-1673-6 (PMC6376702; doi:10.1186/s12870-019-1673-6)
Supplement: Supplementary file 11 — The functionally analyzed proteins of plant species used in this study. (DOCX 13 kb) [file 12870_2019_1673_MOESM11_ESM.docx]

**Additional file 11:** The functionally analyzed proteins of plant species used in this study

| Protein | ID | Resources |
| --- | --- | --- |
| OsD27 | ACT91266.1 | NCBI |
| AtD27 | AT1G03055 | Phytozome V11 |
| Os01g0700900 | BAS73891.1 | NCBI |
| Os01g0701500 | BAS73896.1 | NCBI |
| Os02g0221900 | BAS77710.1 | NCBI |
| Os06g0565100 | BAS98276.1 | NCBI |
| Os01g0701400 | BAS73894.1 | NCBI |
| AtMAX1 | AT2G26170 | Phytozome V11 |
| PhMAX1 | AEB97383.1 | NCBI |
| Os01g0935400 | BAS76087.1 | NCBI |
| LBO | AT3G21420 | Phytozome V11 |
| OsD14 | LOC_Os03g10620 | Phytozome V11 |
| AtD14 | AT3G03990 | Phytozome V11 |
| PhDAD2 | AFR68698.1 | NCBI |
| AtKAI2 | AT4G37470 | Phytozome V11 |
| OsD14L | LOC_Os03g32270 | Phytozome V11 |
| AtMAX2 | AT2G42620 | Phytozome V11 |
| OsD3 | LOC_Os06g06050 | Phytozome V11 |
| PsRAMOSUS4 | ABD67495.1 | NCBI |
| PhMAX2A | AEB97384.1 | NCBI |
| PhMAX2B | AEB97385.1 | NCBI |
| AtAFB2 | AT3G26810 | Phytozome V11 |
| AtAFB3 | AT1G12820 | Phytozome V11 |
| AtEBF1 | AT2G25490 | Phytozome V11 |
| AtEBF2 | AT3G26810 | Phytozome V11 |
| AtVFB1 | AT1G47056 | Phytozome V11 |
| AtVFB4 | AT5G67250 | Phytozome V11 |
| AtSKP2A | AT1G21410 | Phytozome V11 |
| AtCol1 | AT2G39940 | Phytozome V11 |
| D53 | AHC31003.1 | NCBI |
| SMXL6 | AT1G07200 | Phytozome V11 |
| SMXL7 | AT2G29970 | Phytozome V11 |
| SMXL8 | AT2G40130 | Phytozome V11 |
