# Supplementary material for: Identification and expression analysis of strigolactone biosynthetic and signaling genes reveal strigolactones are involved in fruit development of the woodland strawberry (Fragaria vesca)
Source: BMC Plant Biol. 2019 Feb 14;19:73. doi: 10.1186/s12870-019-1673-6 (PMC6376702; doi:10.1186/s12870-019-1673-6)
Supplement: Supplementary file 12 — Primers used in qPCR analysis of this study. (DOCX 13 kb) [file 12870_2019_1673_MOESM12_ESM.docx]

**Additional file 12:** Primers used in this research

| Gene | ID | Purpose | Forward primer (5’-3’) | Reverse primer (3’-5’) |
| --- | --- | --- | --- | --- |
| *GAPDH* | AB363963.1 | qPCR | CATTCATCACCACCGACTACA | GAAGGGTCTTCTCATCCTTGAC |
| *FvD27* | mrna20277 | qPCR | GTGGATTTGAGAGCCTGGTAG | GGGAAAGTATTGGCCTTGGA |
| *FveCCD7* | mrna04863 | qPCR | GCAAGGACACTTCTCCCATATAC | CTTCAATAGGCACTCTCCAATCT |
| *FveCCD8* | mrna08839 | qPCR | GACCGAGTTCCTGACTTTGTT | TCCGATCACCTTCCTTTCATTC |
| *FveMAX1A* | mrna02706 | qPCR | CCATTTGGAATAGGGCCTAGAG | ATGTTTGGAGAGTGCCTGAATA |
| *FveMAX1B* | mrna02708 | qPCR | GCTGCACAGAACTAAGGAAGA | CCCAAGACTCCAGCCAATAC |
| *FveLBO* | mrna06851 | qPCR | GATGTCCAAGAGCTGAGAAAGA | GTGGGAATGTCACTAGAGGATG |
| *FveD14* | mrna02565 | qPCR | TACTTCGATTTCAACCGCTACA | CGACGTAGGCGCACTTT |
| *FveD3* | mrna15755 | qPCR | GCTTTGGGAGAGGTTCTTTCT | TGGTTCACATCCCTGTCTTG |
| *FveD53A* | mrna20979 | qPCR | AAGACGATGGAGTGCTATTGAG | TGCTAAAGGCAAGGGATGAG |
| *FveD53B* | mrna07912 | qPCR | CGGGAAGAATCCCTTACTCATC | GACTAACTTCAGGAGGCAAGAG |
